# Single-Cell Proteomics Reveals Novel Cell Phenotypes in Marfan Mouse Aneurysm

**DOI:** 10.1016/j.mcpro.2026.101549

**Published:** 2026-03-03

**Authors:** Louis Saddic, Ashley Dinh, Giselle Kaneda, Amanda Momenzadeh, Lior Zilberberg, Yang Song, Mitra Mastali, Simion Kreimer, Alexandre Hutton, Ali Haghani, Jesse G. Meyer, Sarah J. Parker

**Affiliations:** 1 Department of Anesthesiology and Perioperative Medicine, David Geffen School of Medicine, University of California, Los Angeles, California, USA; 2 Department of Cardiology, Cedars-Sinai Medical Center, Los Angeles, California, USA; 3 Smidt Heart Institute, Cedars-Sinai Medical Center, Los Angeles, California, USA; 4 Department of Biomedical Sciences, Cedars-Sinai Medical Center, Los Angeles, California, USA; 5 Department of Computational Biomedicine, Cedars-Sinai Medical Center, Los Angeles, California, USA; 6 Board of Governors Innovation Center, Cedars-Sinai Medical Center, Los Angeles, California, USA

**Keywords:** single-cell proteomics, aortic aneurysm, Marfan syndrome, multiomic integration

## Abstract

This report describes single-cell proteomic analyses of cells dissociated from a complex mammalian tissue using direct label-free mass spectrometry (single-cell proteomics by mass spectrometry, SCP-MS). The nanoDTSC approach was applied to profile individual cells from aorta of male and female wild-type and *Fbn1*^*C1041G/+*^ Marfan mice. Leiden clustering identified all major aortic cell types including seven distinct smooth muscle cell (SMC) subtypes, with informative differences in cell proportions and differentially expressed proteins within cell types observed for both genotype and sex. Comparisons between single-cell RNA and single-cell proteomic profiles showed similarities in detection of major subtypes but not differentiation between SMC subtypes. Integrated multiomics analysis further identified genotype-dependent enrichment of unique SMC subtypes, relative to either protein or RNA datasets. Multiplexed-fluorescence based spatial proteomics validated several of these key genotype markers. Overall, these studies demonstrate the power of SCP-MS to detect novel aneurysm biology and serve as a guide for future development of SCP-MS methodology as it is applied to complex tissue cell mixtures and its integration with other omic modalities.

## Highlights

• Single-cell proteomics on nearly 5000 cells from dissociated tissue.
• Biological comparison of cells from aneurysmal and non-aneurysmal tissue
• Novel smooth muscle cell markers and endothelial cell states in aneurysm.
• Multiomic integration comparing proteomic and transcriptomic aneurysm cell states

## Graphical Abstract

**Figure:**
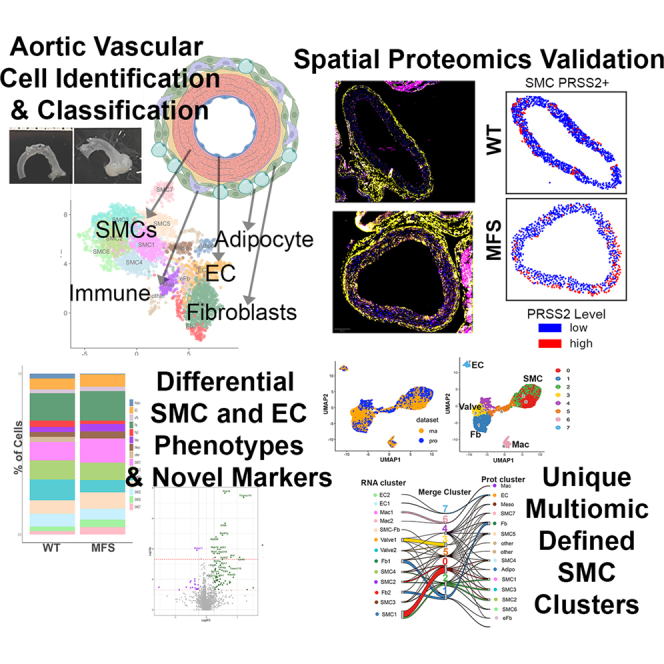

## In Brief

Nearly 5000 cells dissociated from mouse aorta were profiled by direct single-cell proteomics, comparing male and female mice with aneurysm-causing Fbn1 mutation to WT controls. Novel smooth muscle cell type marker Prss2 and the presence of endothelial-to-mesenchymal transition were elevated in cells from aneurysmal Marfan aorta, which was confirmed using spatial proteomics. Comparison of single-cell proteomic to transcriptomic data highlights key differences in cell definitions across modalities, and potentially unique value in multiomic-derived cell phenotyping toward understanding Marfan aneurysm.

The development of single-cell RNA-seq was one of the most consequential recent advancements to the field of genomics. It allows unbiased characterization of individual cells using transcriptomic profiles in a way that has broadened our understanding of tissue diversity and cellular plasticity (1). Transcripts are often taken as a proxy for protein abundance and expression, but due to numerous levels of biological regulation (*e*.*g*.*,* RNA stability, protein translation rates, and protein degradation rates), the correlation between RNA and protein expression can be variable from one gene to the next (2, 3, 4, 5, 6). Therefore, there is a strong interest in developing single-cell proteomic technology to complement and extend cell phenotype analysis gleaned from transcriptomic profiling (7).

Initial applications of single-cell proteomics utilized very large *xenopus* embryo cells, each with adequate protein content for detection on earlier generation mass spectrometers (8). A leap in capability occurred with the development of the single-cell proteomics by mass spectrometry (SCoPE-MS) approach, which uses tandem mass tag (TMT) peptide labeling of individual cell peptides and multiplexes several cells into a single MS run, all alongside a “carrier” channel of labeled peptides from a representative bulk lysate. While this enabled *bona fide* single-cell profiling, issues with TMT have led researchers to explore alternatives. The main challenges with TMT-based single-cell proteomics are ratio compression and coisolation interference, which distort quantitative accuracy due to overlapping precursor ions in multiplexed samples. In addition, ion suppression and sample complexity from high carrier loads reduce sensitivity for low-abundance proteins, while high reagent costs and sample complexity constrain scalability compared to data-independent acquisition label-free quantification (DIA-LFQ), which offers improved quantification and dynamic range.

Direct label-free single-cell proteomics analysis overcomes many of these issues, which was recently made possible through the design of an ultrasensitive mass spectrometer, the Bruker timsTOF SCP instrument (9). Alongside advancements in MS sensitivity, others have optimized nanoscale sample preparation, and our group and others have advanced liquid chromatography (LC) methods to enable fast, sensitive separation of peptides in-line with MS to improve the throughput (*e*.*g*.*,* number of cells profiled per 24 h) (10). All together, these advancements have launched LC MS based proteomics onto the map of options for single-cell profiling, with a steady release of recent publications highlighting novel and complementary discoveries made by this technology (11). To date, nearly all single-cell proteomic studies published have focused on *in vitro* cell systems with moderate heterogeneity, raising the question of how the technology and data analysis approaches will perform on cellularly complex organ systems.

In the current study, Marfan’s syndrome (MFS) was chosen as a model system to push the limits of current single-cell proteomics by mass spectrometry (SCP-MS). MFS is an autosomal dominant genetic disorder resulting from mutations in the fibrillin 1 (*Fbn1*) gene. Fbn1 encodes an extracellular matrix (ECM) protein that is a key component of elastic-microfibrils that support distensibility, elasticity, and compliance, and also binds and modulates several growth factors in tissues such as the vasculature (12). Given its prominent ECM distribution, Fbn1 interacts with most cells of the arterial wall and adventitia, thus disease-causing mutations can elicit unique effects by vascular cell type. Patients with MFS develop phenotypic alterations in many organ systems but perhaps the most deleterious is aneurysms of the aortic root that carry a high risk of dissection and sudden death. In these patients, there is aberrant destruction of the medial layer of arteries leading to aneurysm growth of the aortic root and ascending aorta. Mouse models of MFS, including the *Fbn1*^*C1041G/+*^ mouse, recapitulate the aortic disease found in humans, and have been invaluable to uncovering the molecular causes that lead to this disease (13). Despite decades of knowing and researching the causal mutations driving MFS aneurysm, this has not translated to an impactful enough drug treatment to slow or stop aneurysm progression and as of now, the only treatment to Marfan aortic root aneurysms is high-risk cardiac surgery (14, 15, 16, 17). Part of the knowledge gap relates to an ongoing lack of clarity as to how MFS-causing Fbn1 mutations drive aneurysm progression and dissection, and it is likely that mapping cell-type specific responses to affected tissue ECMs harboring the Fbn1 mutation within the *in vivo* system is needed to compile a clear understanding of molecular pathogenesis. Identifying the integrated response of cells to the Fbn1 mutation, and the causes and identity of cellular phenotype shifts could be the key to reversing disease progression.

Our group recently developed a workflow for high-throughput direct single cell profiling of up to 96 cells per day, which enabled proof of concept SCP-MS profiling on 1 mouse aorta, representing one of the first complex tissues to be analyzed by this new platform (10). We demonstrate here that SCP-MS can accurately profile individual cell types in mouse aortic root tissue and further subdivide SMCs into distinct subgroup populations. In addition, we show that this new technology can uncover novel biological differences in the abundance of cell types as well as cell specific differentially expressed proteins (DEPs) based on sex and the MFS phenotype, including evidence of potential endothelial-to-mesenchymal transition (EndMT) in MFS. Even more, we show that combining single cell RNA with single-cell proteomic profiles of Marfan mouse aortic roots can provide a complementary lens into modified SMC phenotypes that may play a role in aneurysm biology.

## Experimental Procedures

### Experimental Design and Statistical Rationale

The study design cells from N = 3 biological replicates of four experimental groups (male WT, female WT, male MFS, and female MFS). Batch correction by sample plate was used for unbiased Leiden-based clustering for the purpose of assigning cells to a given phenotype based on their proteomic data in order to minimize segregation by technical rather than cell identity factors. Linear mixed-effects models were used to compare protein abundance by sex and genotype within a given cell type cluster (see additional details, below). Using scPower as an approximation for pairwise differential testing within a given cell type, estimating a cell frequency of 0.13 per mouse among the ∼290 quality control filtered cells measured per animal, three mice per group yields an estimated power of 0.385 and pooling sex or genotype to achieve N = 6 yields a power of 0.457.

### Isolation of Mouse Aortic Root Cells and Processing for Proteomic Analysis

All procedures involving mice were approved by the Cedars-Sinai IACUC review panel (Protocol #IACUC008535). N = 3 biological replicates of aortic root cell suspensions were produced per genotype-sex group (male WT, female WT, male MFS, female MFS, and total distinct samples N = 12), as described in Supplemental Methods. The cell suspensions, containing approximately 3 to 5 x 10^4^ cells in 200 μl, were labeled for viability with SyTox green dye and transferred into the loading vial of a Cellenion cellenONE single-cell isolation and robotic liquid handling instrument (version F1.4, Software version 2.0). Cells ranging in diameter from 10–50 μm with minimal green fluorescence (*e*.*g*.*,* live cells) were dispensed one per well of a 384-well plate that was preloaded with 200 nl of lysis buffer (100 mM TEAB, 0.2% DDM in 50 mM Tris pH 8.0). Plates of sorted, lysed cells were stored no more than 60 days, covered at −80 °C until further processing. Lysates were digested by dispensing 200 nl of 40 μg/ml trypsin into each plate well (final of 8 ng trypsin per cell, digestion buffer pH between 7 and 8), and incubated for 4 h at 37 °C before quenching digestion with 200 nl of 0.1% formic acid solution. Quenched digested peptide was dried completely, and plates were placed on an autosampler for liquid chromatography mass spectrometry analysis.

### Direct Single-Cell Proteomic Data Acquisition, Peptide Sequence Identification, and Protein Quantification Estimation

LC-MS was based on the nano dual trap single column (nanoDTSC) method described in Kreimer *et al* (10), with detailed methods provided in Supplementary Text. Raw files were searched using DIANN v1.8.1 (18) against the custom assembled sample specific library (See Supplementary Text, Supplemental Fig. S1, and Supplementary Table “Final Library”), using match between runs and reannotation of gene groups to a supplied mouse FASTA protein sequence database, but with no heuristic protein inference and no normalization applied. Protein quantification estimates were produced using the DIANN maxLFQ algorithm, and only proteins quantified by at least one proteotypic peptide were included in the final dataset for further analysis.

### Bioinformatic Analysis of Single-Cell Proteomic Data

Data QC and analysis were performed using the Single-Cell Analysis in Python (Scanpy) python package (19), which has been reported on previously for use in processing, visualizing, and analyzing SCP-MS (20, 21, 22) and TMT-based SCoPE2-MS (23) datasets. The final protein-by-cell data matrix was uploaded and converted into an annotated data matrix object, with unobserved or quantified proteins annotated as zero. Cells with fewer than 200 proteins identified and proteins observed in fewer than N = 3 cells were dropped before normalizing using the Scanpy normalize_total function, which normalizes each protein in a cell to the total sum of all protein intensities for that cell, and then scales each measured intensity by multiplying each proteins normalized intensity by a cell-specific factor calculated by dividing the summed intensities for that cell by a target sum, in this case 1e (4) (see package documentation for more details (19)). Protein intensities were then transformed to log scale using the log1p function (log_10_(1+x), where x = a given protein intensity). Principal component analysis (PCA) at this point indicated that the protein identification count per cell, annotated as “n_genes” by scanpy, varied noticeably along PC1 (Supplemental Fig. S3*C*). To minimize sampling depth as a driver of variance in downstream analysis, a linear regression adjustment was applied to adjust a given cells’ protein intensities by the total number of proteins observed for each cell using the “regress_out” function, which replaces values with residuals after removing the linear effect of a designated covariate, in this case the total number of proteins identified in a given cell (annotated as “n_genes”). Batch effect correction was performed using ComBat (24, 25), with each plate considered a separate batch, and data matrix completion was achieved by use of zero (*versus* NA) for missing proteins in a given cell. Batch corrected protein matrices were then processed using PCA applied using all proteins in the dataset after preprocessing, from which cell neighborhood calculations were performed ahead of leiden based clustering (using default parameters except resolution which was set to 1.2). Finally, uniform manifold projection (UMAP) dimensionality reduction and visualization was conducted. In order to explore possible plasma-protein or cell-stress protein distributions, the high abundant plasma proteins albumin (alb) and two hemoglobin subunits (Hbb-1 and Hbb-2) were quantified across batches. For cell stress, a composite score of heat shock protein (HSP) intensities was summed from all HSP detections in a given cell. General cell type annotation was deciphered using canonical marker proteins, with unbiased marker analysis performed for corroboration as well as identification of cell subtype markers. Cell proportions were compared between groups at the level of all observed cells per group, and all cells observed per biological replicate. Figures were generated using Scanpy or Suerat after exporting the Scanpy annotated data matrix object and converting it into a Seurat object for additional figure generation and analysis in R (26).

### Differential Protein Analysis and FDR Control

Differential expression (DE) testing was performed only on non-ComBat corrected data, since batch correction was heavily affected by experimental group (*e*.*g*.*,* sex, genotype). Thus, after its use to identify cell neighborhoods, clusters, and make predictions of their biological phenotypes, we did not use batch corrected data for the intracell type DE analysis. The DE was performed separately for cells within each leiden assigned cluster designation (*e*.*g*.*,* within each cell phenotype). For each protein we fit a linear (mixed-effects) model with fixed effects for group/sex and a random intercept for mouse (inference on the group/sex coefficient). Models used log1p intensities from cells with a nonzero measurement for the protein and required ≥3 cells per group to enter testing. *p* values were adjusted across M tested proteins using Benjamini–Hochberg false discovery rate (FDR) at q = 0.05.

### Comparison of Single-Cell Proteomic Data to a Similar Published Single-Cell RNA Sequencing Dataset

Raw counts were downloaded from the Gene Expression Omnibus database using the identifier GSE186845 belonging to the datasets described in Pedroza *et*. *al*. from 16-week-old WT and MFS *Fbn1*^*C1041G*^ male and female mice (N = 1 per group) (27). Single cell transcriptomic (scTranscriptomic) data were reanalyzed using the same Scanpy workflow used to analyze the protein data, with the exception that batch effect correction was not performed. A total of 8000 genes were analyzed across 39,031 individual cells. Marker genes either matching the canonical SCP-MS cell type marker set or derived from the original source publication were used to ascertain cell-type cluster identifications, with unbiased marker analysis also being performed. The scTranscriptomic and SCP-MS data objects were then exported and converted to separate Seurat objects in R using the Schard package (https://github.com/cellgeni/schard). An initial comparison of the two datasets was performed using the Seurat package “FindTransferAnchors” and “TransferData” functionality (28), in which shared marker proteins from one data object are used to predict cell types to the other dataset for comparison of how well the molecular patterns in one dataset translate to subsetting cell types in another dataset (additional details in supplementary text). In order to further compare the datasets, we performed a random downsampling of the scRNA dataset to match the proteomic dataset size more closely, selecting a random subset of 5000 cells for downstream integrated analysis. We then used the LIGER (29) multi-omic integration package in CRAN (30) to merge the count matrices from the proteomic and downsampled RNA datasets, with k = 10 and lambda = 5 used for the “runIntegration” merging step. The merged LIGER object was quantile normalized after which Leiden clustering (nNeighbors = 30, resolution = 0.5) and UMAP dimensionality reduction (nNeighbors = 30, minDist = 0.3) were performed. Canonical marker genes/proteins were used to estimate cell type annotations onto new, integrated LIGER Leiden clusters. A Sankey plot was constructed comparing the groupings of any given cell’s annotation between their parental dataset (*e*.*g*.*,* RNA or protein only) and the integrated, LIGER dataset. Next, we selected only the subsets of assigned SMC Leiden clusters from each dataset (scRNA and SCP-MS, based on the prior assignment from each individual analyte type experiment), again applying a random down sampling on the RNA dataset to include only 2200 SMCs, comparing more equivalently to the 2031 SMCs subset from the SCP-MS dataset. The same liger integration steps were then performed on the SMC-specific cells, including Leiden cluster assignment, UMAP projection, and Sankey plot comparison to the original cell cluster assignment from the original single-analyte analysis. In addition, for the SMC-specific analysis, the proportion of cells from each genotype, whether from RNA or protein datasets, were calculated across the new LIGER clusters. To assess whether the proportions of sex (male *versus* female) and genotype (MFS *versus* WT) differed significantly across clusters, we performed two-sided z-tests for proportions. First, the dataset was reformatted into a long format, where each observation contained the LIGER cluster assignment, sex, genotype, and count. We computed the overall proportions of females and MFS samples across all clusters as reference values. For each cluster, we compared its sex-specific and genotype-specific proportions to the overall dataset proportions using a z-test for two proportions, which tests whether the proportion in a specific cluster significantly deviates from the expected proportion under the null hypothesis of no difference. Finally, we analyzed marker features (shared protein and transcript observations) across the LIGER Leiden clusters using its built-in Wilcoxon test of expression in the test cluster relative to all other clusters. Kyoto Encyclopedia of Genes and Genomes (KEGG) pathway enrichment (31) of the top 100 shared marker features with Log_2_FC > 1 for a given cluster expression was analyzed in the ENRICHR platform (32).

### Spatial Proteomic Validation Experiment

To prepare for a multiplex immunofluorescence experiment, tissue slides from FFPE proximal aorta from N = 4 male mice (N = 2 WT, N = 2 MFS, age 12 weeks at sacrifice) were baked at 60 °C for 90 min followed by immersion into xylene for 20 min for deparaffinization. Slides were then immersed in HIER H (Epredia,) antigen retrieval buffer solution for 1 h at 98 ^o^C and cooled to room temperature for an additional 30 min. After washing in Multistaining Buffer (Lunaphore Technologies) photobleaching was performed by two incubations in 3% Hydrogen Peroxide solution under an LED lamp for 40 min at room temperature. The slides were then loaded into the Lunaphore COMET instrument for multiplex sequential immunofluorescence (seqIF) staining and image acquisition. A 21-cycle seqIF protocol was built into the COMET software based on a previously published approach (33) in order to enable acquisition of IF data on previously validated antibodies, summarized in Supplemental Table S1. The first cycle images detect autofluorescence of the tissue for background subtraction pre-analysis. Subsequent successive cycles consist of a 16 min primary antibody incubation, a 4 min secondary antibody incubation, imaging, and a final 4-min elution step. At the completion of the 21-cycle protocol, images were stitched and viewed with the HORIZON ViewerTM where background subtraction was performed and stacked OME-TIFF files were exported for further analysis. Multiplexed image analysis was conducted in QPATH v0.6.0-rc4 (34). An ROI was traced around the exterior border of the aortic segment, encompassing the adventitia and perivascular regions but excluding any nearby adjacent vessels or myocardium. The built in StarDist extension was used for cell segmentation, based on DAPI nuclei detection and expansion to estimate cell borders. The mean intensity of each fluorescent channel for each of the detected cells in each section were exported to a tab-separated file. Using the python SCIMAP package (35), signal intensities from each channel were log transformed and then scaled using the SCIMAP ‘rescale’ function using individual gates set based on the mean intensity of a given channel across all cells identified in a given tissue section. To enable more direct comparison between SCP-MS and spatial proteomic data, we applied the same scaling approach to the selected SCP-MS proteins to which spatial data were compared (*e*.*g*.*,* Prss2, Lrp1, Ace, Tpm4, and S100a4). Specifically, the mean value for a given protein across all cells, regardless of cell type, in a batch was used as the manual gating file and the observations of each protein of interest in the SCP-MS dataset were also run through the “rescale” function in SCIMAP to set intensities between 0 to 1, enabling comparable calls between the two datasets for “positive” (*e*.*g*.*,* scaled intensity >0.5) and “negative” cells for a given marker. After scaling, principle cell phenotypes were assigned according to the curated marker types and include the following: EC (Pecam1), SMC (Acta2), Fibroblasts (S100a4), and myeloid (CD11b) cells. SMAD2/Zeb1 positivity in addition to platelet endothelial cell adhesion molecule 1 (Pecam1) was used to assign the EndMTcell type and ACE/TPM4 or S100a4 positivity in addition to Acta2 was used to assign the multiomic SMC subtypes. Cell proportions and abundance of other markers were then assessed using built in SCIMAP graphing and analytical functions.

## Results

### Single-Cell Protein Quantification and Quality Control Results Across Mouse Aortic Cells

**Fig. 1:**
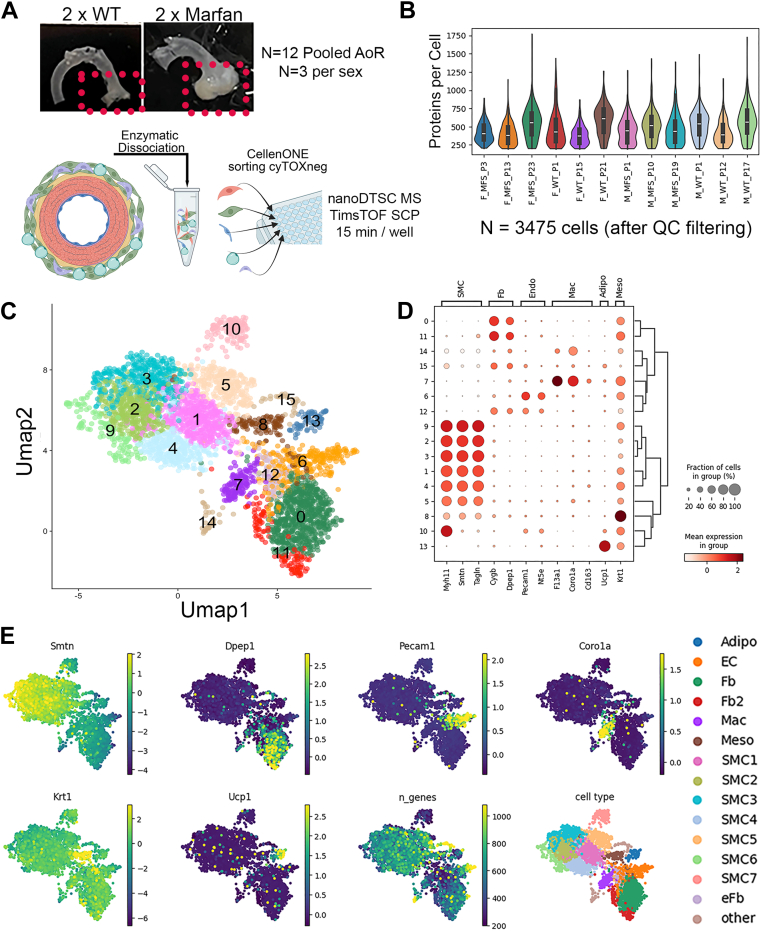
**Experimental workflow and results overview**. *A*, overview of the experimental workflow. Two aortae (*top panel*) from male or female, wild-type (WT) or *Fbn1*^*C1041G*^ Marfan’s syndrome (MFS) mice were enzymatically dissociated to release individual cells, which were subsequently pooled sorted on a CellenONE system into 384-well plates for lysis and tryptic digestion ahead of acquisition of individual cell proteomes on the Bruker TIMSTOF SCP mass spectrometer using the nanoDTSC workflow (N = 12 biological replicates, N = 3 per sex/genotype combination). *B*, distribution of proteins identified per cell, separated by processing batch. *C*, projection of all mouse aortic cells onto UMAP dimensionality reduced space, with 16 sets of proteomically-similar cells (*e*.*g*.*,* cell types) mapped as separate colors using leiden algorithm. *D*, Dot plot to display the expression percentage and average intensity of canonical cell type markers for known aortic cells across the 16 identified Leiden clusters. *E*, cell phenotype assignments based on canonical expression patterns. Fbn1, fibrillin 1; nanoDTSC, nano dual trap single column; UMAP, uniform manifold projection.

The overall study design is summarized in Figure 1*A*. The majority of cells dispensed were <20 μm in diameter (Supplemental Fig. S2*A*) with average cell diameters by batch within 2 μm range across all plates. There were no significant differences in the mean cell diameters across sex and genotype combinations (Supplemental Fig. S2*B*, *p* = 0.726, one-way anova). As expected, cell diameter correlated positively with both total MS1 signal (Supplemental Fig. S2*C*) and number of proteins identified (Supplemental Fig. S2*D*). Cells with the lowest protein identification numbers tended to have higher estimated missed tryptic cleavages (Supplemental Fig. S2*D*), suggesting sub-optimal digestion as a significant contributor to lower protein identifications. A total of 2746 protein groups were detected from 157,773 peptides across 4986 cells (Protein and Peptide level data provided in Supplemental Tables S2 and S3, respectively). The proportion of empty wells, low protein count, and included cells is shown in Supplemental Figure S3*A* for each batch. The detection of zero signal runs of presumably empty wells spaced variably throughout the acquisition schema (data not shown), coupled with almost all intentionally run blank samples yielding zero protein identifications (Supplemental Fig. S3*B*) supported low to no carryover from run to run. Given that the highest protein count from any individual blank run was <125, we set a conservative cut-off of 200 proteins per cell as a lower limit for inclusion of a cell in subsequent analysis. After filtering to remove cells with <200 proteins identified, removing non-proteotypic identifications (*e*.*g*.*,* protein groups identified by shared peptide sequences) and proteins seen in fewer than 3 cells across the dataset, a total of 3475 cells and 2421 proteotypic (*e*.*g*.*,* quantified by only unique peptide sequences) proteins were available for downstream analysis (Supplemental Table S4), with an average of 487 identified proteins identified per cell, distributed evenly across biological replicates and plates (Fig. 1*B*). In preliminary PCA we observed a trend for number of protein identifications per cell driving PC1 (Supplemental Fig. S3*C*, left), and thus applied linear regression on total number of proteins identified (annotated as “n_genes”) to mitigate this effect as a potential factor driving cell clustering (Supplemental Fig. S3*C*, right). As expected, normalization and preprocessing substantially improved the overall variability range, as indicated by the distribution of protein intensity coefficient of variation (CV) across all cells and within the cells of each plate batch (Supplemental Fig. S3, *D* and *E* for non-normalized and normalized coefficient of variation distributions, respectively). After initial dimensionality reduction and clustering, we observed uneven mixing of batches and biological replicates across clusters (Supplemental Fig. S4*A*), prompting batch correction using ComBat (24) correcting on cell processing plate batch. This ensured that all biological replicates were well balanced across most cell clusters (Supplemental Fig. S4*B*). Importantly, batch effects were improved when also considering distribution of Leiden clusters within a given plate batch (Supplemental Fig. S4*C*). Proteins that are likely derived from blood contamination (albumin, hemoglobin subunits Hbb-1 and 2) were detected in some cells but did not appear to drive Leiden clustering, and likewise heat shock protein score was not substantially different by batch or most Leiden clusters (Supplemental Fig. S5*A*). When compared to bulk proteomic analysis, these contaminant or stress proteins were generally lower in relative rank (*e*.*g*.*,* intensity relative to the median intensity across all aortic samples (bulk data) or measured cells (SCPMC data)), indicating that the dissociation technique generated relatively clean cell preparations with no overt indicators of cell stress caused by the dissociation, at least in terms of HSP score.

### SCP-MS Identifies Major Aortic Cell Subtypes

Leiden clustering predicted 16 cell groups. Plasma proteins albumin and hemoglobin were detected with variable amount across the isolation plate batches, but did not appear to substantially drive Leiden clustering (Supplemental Fig. S5). The HSP composite score was used as a proxy for cell stress, and showed no major differences by isolation batch, albeit some enrichment in the smallest two Leiden clusters (Supplemental Fig. S5, lower left and right panel) was observed. Analysis of specific marker gene expression, selected to represent well known aortic cell types, was then performed across the Leiden clusters (Fig. 1*C*). A distinct pattern of canonical aortic cell phenotypes emerged, based on reciprocal abundance patterns of these markers. Specifically, we observed that six Leiden clusters (1, 2, 3, 4, 5, and 9) demonstrated strong and consistent expression of SMC markers (smoothelin (Smtn), transgelin (Tagln), and myosin heavy chain 11 (Myh11)) with a seventh having high smooth muscle specific myosin (Myh11) but low abundance of other SMC markers (cluster 10). We also observed two clusters (clusters 0 and 11) with strong fibroblast marker abundance (dipeptidase 1 [Dpep1], and cytoglobin B [CygB]), and two (clusters 6, 12) with strong endothelial cell (EC) marker expression Pecam1 and ecto-5′-nucleotidase/CD73 (Nt5e)), one of which also had notable fibroblast marker levels (*e*.*g*.*,* cluster 12). Macrophage markers (Coronin 1a (Coro1a) and Factor 13a1 (F13a1)) identified one strong cluster (cluster 7) and were marginally also present in cluster 14. Cluster 8 was high in the epithelial markers, including Keratin 1 (Krt1), and was annotated as mesothelial. Interestingly, cluster 13 had high uncoupling protein 1 (Ucp1) levels, which is consistent with a perivascular adipose cell phenotype (36). In addition to these *a priori* selected markers, the top 15 marker proteins identified using unbiased statistical comparisons for each cluster are plotted in Supplemental Fig. S6 and Supplemental Fig. S5. To further assess clusters and their putative cell types, cell proteomes were then visualized in dimensionality-reduced space determined by UMAP (Fig. 1*D*), where the clusters with strong SMC markers aggregated together on the left portion of the graph, while all other cell types were distributed on the right portion, with cell types of similar canonical marker expression clustering closely together in UMAP defined 2-dimensional space (Fig. 1*E*). Projection of canonical markers directly on to PCA plots also exhibited a clear separation between SMC and the other main cell types, but failed to distinguish as well between fibroblasts, adipocytes, ECs, and macrophages with the same clarity as the UMAP visualizations (Supplemental Fig. S7). In summary, the pre-processing approach and analytical strategy applied resulted in a set of defined cell clusters which matched well characterized aortic phenotypes, with sufficient sampling of cells to reveal sub-clustering within some of these broad cell type categories (*e*.*g*.*,* SMCs and fibroblasts).

### Unique Proteome Signatures Define Smooth Muscle Cell Subtypes

**Fig. 2:**
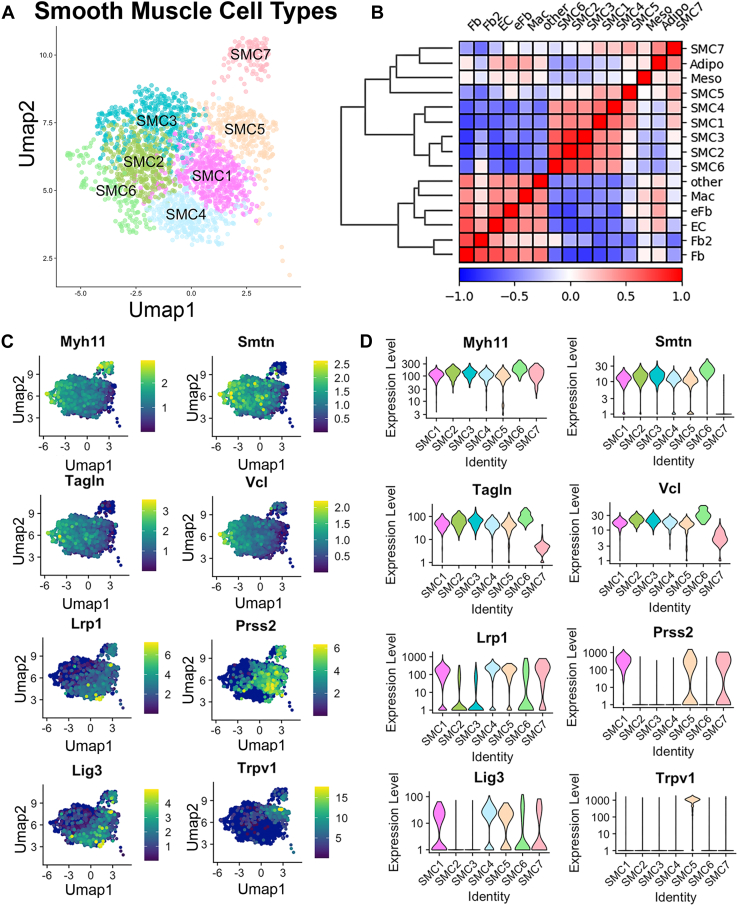
**Deeper analysis of smooth muscle cell (SMC) subphenotypes**. *A*, UMAP projection of only SMC–cell sub phenotypes (SMC1-SMC7). *B*, correlogram of proteomic similarity between and across all cell types identified. *C*, projection of identified SMC–subtype marker proteins. *D*, violin plots of quantified marker expression levels across SMC subphenotypes. UMAP, uniform manifold projection.

Not surprisingly, SMCs were the predominant cell type from aortic samples, accounting for 58.4% of all cells when cell counts across all SMC subtypes (leiden 1, 2, 3, 4, 5, 9, and 10 selected out by leiden assignment and projected by genotype and sex in Fig. 2*A*) were aggregated. When examining the correlation between global proteomes of all cell types in the aorta, all SMC subtypes were most similar to other SMC subtypes with the exception of SMC subtype 7 (corresponding to Leiden cluster 10), which shared more similarity with adipocytes (Fig. 2*B*). SMC7 also stood out in terms of cell diameter, in that while all other clusters of SMCs had similar distribution of diameters, centering around 19 μm, SMC7 was noticeably smaller in diameter (Supplemental Fig. S8). SMC7 was designated an SMC due to its high abundance of Myh11 despite low expression of other SMC markers such as Smtn and Tagln (Fig. 2, *C* and *D*). When we examined some of the protein abundance patterns among the different subtypes of SMCs (Fig. 2*D*) a few relationships emerged. Along the UMAP axis, SMC2, SMC3, and SM6 occupied the leftward region of the SMC UMAP space and were in general classified as having somewhat higher abundance of the known canonical SMC markers, namely Myh11, Smtn, Tagln, and vinculin (Vcl). On the other hand, SMC1, SMC4, SMC5, and SMC7, occupying the rightward SMC UMAP space, had slightly lower abundance of these canonical markers of SMCs, leading to a broad classification of these two sets as “contractile” (SMC2, 3 and 6) *versus* “modified” (SMC1, 4, 5, and 7) phenotypes. Among the putative “modified” clusters, we observed some intriguing novel potential marker proteins of this SMC phenotype (Fig. 2, *C* and *D*), with higher abundance of the proteins low-density lipoprotein receptor-related protein (Lrp1), protease serine 2 (Prss2), and DNA ligase 3 (Lig3). Of note, Prss2 appeared more selective for just SMC1, 5 and 7. Furthermore, SMC5 had uniquely high abundance of the capsaicin channel transient receptor potential vanilloid 1 (Trpv1).

### Sex and Genotype Alter Abundance of Cell Types

**Fig. 3:**
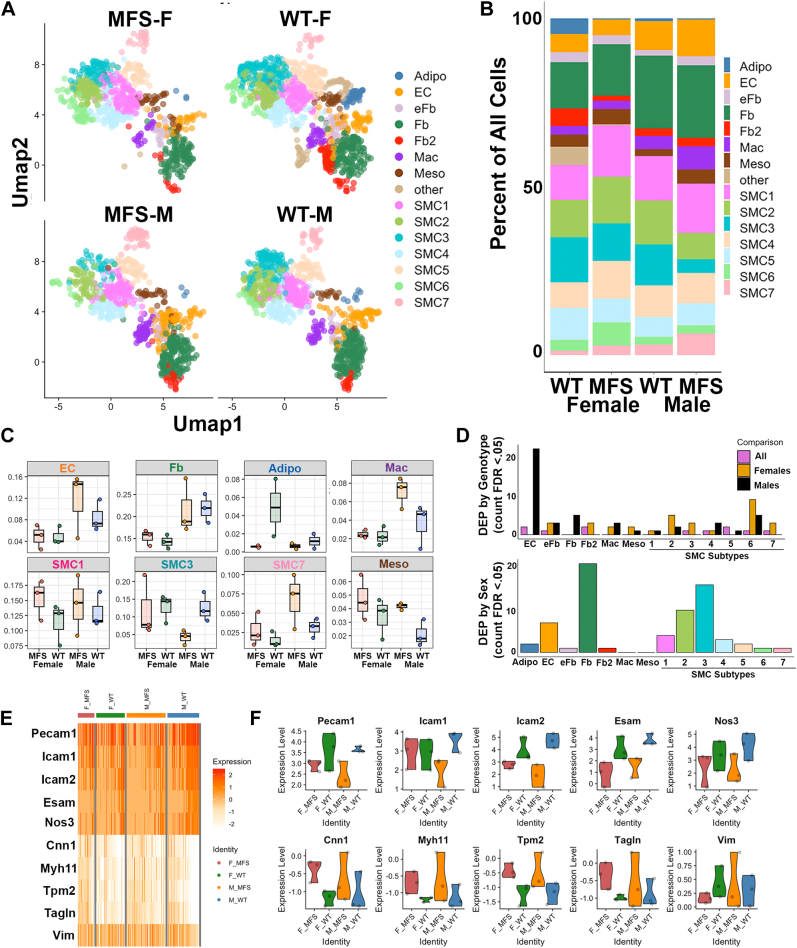
**Comparison of sex and genotype patterns across single-cell proteomic data**. *A*, UMAP projection of aortic cell types separated by donor mouse sex and genotype. *B*, relative proportion of each assigned cell type across all cells identified per mouse sex and genotype category. (*C*) Selected proportions of a selected cell type based on percentage assigned within an individual mouse biological replicate (N = 3 biological replicates per sex and genotype combination). (*D*) Count of significant differentially expressed proteins (FDR <0.05) from linear mixed-effects modeling comparing genotype (*upper panel*) or sex (*lower panel*) within each assigned cell type. For genotype, effect of both sexes combined was compared to differentially expressed proteins (DEPs) within males (M) and females (*F*) (by genotype) separately. *E*, subset of key proteins potentially indicative of endothelial-to-mesenchymal (endMT) transition with expression intensity in Marfan’s syndrome (MFS) *versus* wild-type (WT) endothelial cells (ECs). *F*, violin plots of endMT indicators across male and female MFS and WT endothelial cells. UMAP, uniform manifold projection.

Cells from each experimental category (n = 3) were pooled and the corresponding UMAPs and relative abundance of each cell type within each sex and genotype combination were plotted (Fig. 3, *A*–*C*). The observed proportion of cell types within a given biological replicate were also quantified (Supplemental Table S6) and compared by experimental category. Male mice exhibited a higher relative proportion of ECs (10.0% *versus* 4.9% of all cells, *p* value 0.02), macrophages (5.3% *versus* 2.4%, *p* value 0.023), and fibroblasts (21.7% *versus* 14.8%, *p* value 0.0049), but not SMCs, compared to female mice regardless of genotype (N = 6 observations per group, genotypes combined). Genotype-related cell proportions, on the other hand, showed sex-differences in cell type abundance wherein the male mice had significantly fewer of the putative “contractile” SMC3 cell type (12.5% of all cells in WT *versus* 4.2% in MFS, *p* value 0.04, N = 3 male WT *versus* male MFS), significantly more of the mesothelial cell type (4.2% of all cells in MFS *versus* 2.1% in WT, *p* value 0.02), and trended toward relatively higher proportions of macrophages (7% in MFS *versus* 3.6% in WT, *p* value 0.1) and putative “modified” SMC cluster SMC7 (6.8% in MFS *versus* 3.1% in WT, *p* value 0.17). These differences were not observed for the female MFS mice relative to their WT counterparts. The putative “modified” SMC1 cluster trended toward upregulation in both female and male MFS aorta compared to WT (14.8% in MFS *versus* 12.2 in WT, *p* = 0.22).

### Differential Protein Expression by Sex and Genotype Within Leiden-Specified Cell Types

Next, the number of DEPs between WT and Marfan mice and between male and female mice within each cell type was compared using linear mixed-effects modeling and an FDR cutoff of 0.05 (Full DEP outputs in Supplemental Tables S7–S10). Overall, there were relatively few proteins in each cluster that met the FDR cutoff for either set of comparisons (Fig. 3*D*), whereas notably larger numbers of possible DEPs were apparent by nominal *p* values (Supplemental Fig. S9*B*), consistent with the estimate of low statistical power. Relative to the other identified cell types, there were noticeably higher numbers of sex specific DEPs in fibroblasts (N = 6 per group, ignoring genotype), with reasonably balanced DEPs upregulated in males *versus* females when visualized by volcano plot (Supplemental Fig. S10*A*). For genotype, we examined overall effect (N = 6 pooled male and female per group) as well as within-sex (N = 3 of one sex, MFS *versus* WT) genotype-driven differences by cell type. As with the sex DEPs, a relatively small number of proteins met FDR cutoffs to be confidently considered DEPs (Fig. 3*D* and Supplemental Fig. S9*A*). Interestingly, there were noticeably more DEPs by genotype in ECs, particularly in male mice. Visualization of EC-specific genotype driven DEPs using volcano plots further corroborated this observation, and also indicated trends for reasonable DEP levels overall as well as within female comparisons in addition to just the male ECs (Supplemental Fig. S10*B*). When we further analyzed the identity of the DEPs in MFS *versus* WT ECs, we noticed that they represented patterns that could be associated with the process of EndMT, namely reduction in EC markers and upregulation of mesenchymal markers. Male Marfan mice had particularly lower expression of endothelial adhesion proteins such as Pecam1, intercellular adhesion molecule 1 (Icam1), intercellular adhesion molecule 2 (Icam2), and EC-selective adhesion molecule (Esam) along with higher expression of smooth muscle proteins such as calponin 1 (Cnn1), Myh11, Tagln, and vimentin (Vim) indicative of a mesenchymal like state (Fig. 3, *E* and *F*), with levels of these proteins also altered in female MFS ECs. To further corroborate this finding, we performed an analysis of EC types profiled in a previously published single-cell transcriptomic data set from *Fbn1*^*C1041G/+*^ mice, and found that mRNA for two transcriptional regulators of endMT, zinc finger E-box-binding homeobox 1 (Zeb1) and snail family transcriptional repressor 1 (Snai1), were elevated in both male and female MFS ECs relative to WT (Supplemental Fig. S11), providing external corroborating support for potential endMT in MFS.

### Validation of Single-Cell Proportions, Selected Markers, and EndMT Phenotype Using Spatial Proteomics

**Fig. 4:**
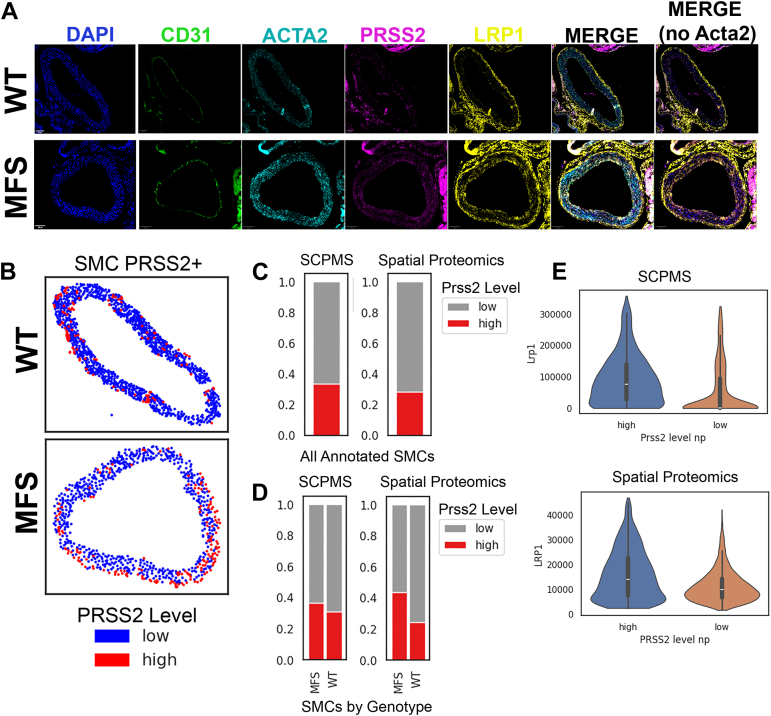
**Validation of Prss2 and Lrp1 by spatial proteomics**. *A*, individual images showing immunofluorescence signal of selected markers in representative WT and MFS sections. Two merged images are shown, one with and one without Acta2 general smooth muscle marker in order to aid visualization of Prss2 and Lrp1 double positive smooth muscle cells. *B*, dot plots from two representative sections showing the centroid x and y coordinates of each smooth muscle cell in the spatial proteomics analysis, with dot color reflecting level of Prss2 signal from a given SMC. *C*, proportion of Prss2 high cells among all SMCs as measured in the SCP-MS and spatial proteomics experiments. *D*, proportion of Prss2-high SMCs between MFS and WT genotypes as measured in SCP-MS and spatial proteomics experiments. *E*, distribution of Lrp1 intensity in cells separated by high or low Prss2-levels in SCP-MS *versus* spatial proteomics datasets. MFS, Marfan’s syndrome; SCP-MS, single-cell proteomics by mass spectrometry; SMC, smooth muscle cell.

In order to confirm the validity of some of our key observations from SCP-MS analysis, we assembled an antibody panel including several of the more novel markers and used cyclic fluorescence-based imaging to generate a spatial proteomic dataset for N = 4 male aorta (N = 2 each of MFS and WT). After cell segmentation and phenotype assignment, we quantified the proportion of key cell types and selected marker expression patterns and compared these to corresponding SCP-MS estimates. Overall, the spatial proteomics workflow resulted in a spatial pattern of cell type assignments consistent with expected anatomy of a typical mouse aorta, with ECs occupying the intimal layer, SMCs dominating the medial layer, and fibroblasts and most immune-like cells occupying the adventitial segment (Supplemental Fig. S12, left panel, color coded cell-type assignment for each centroid x and y coordinate of a predicted cell after segmentation analysis). Using the spatial proteomics data, we examined the novel SCP-MS finding of Prss2 as a marker of the modified SMC phenotype (Fig. 4*A*). Prss2^high^ cells were observed among the SMC cell types within the medial wall (Fig. 4*B*, each dot showing centroided x and y axis coordinate of an SMC assigned, segmentation predicted cell with red dots indicating Prss2-high SMCs), and the proportion of Prss2^high^ cells was nearly identical between SCP-MS and spatial proteomic estimates (Fig. 4*C*). Prss2 was more prominently expressed in the SMC1, 5 and 7 subtypes in the SCP-MS data, two of which showed trends for upregulation in MFS relative to WT. Accordingly, we observed upregulation of the proportion of Prss2^high^ SMCs in MFS aorta relative to WT in both the SCP-MS and spatial proteomic datasets (Fig. 4*D*). Lrp1 was another putative modified SMC marker and showed expected higher signal in Prss2^high^ *versus* Prss2^low^ SMCs in both the SCP-MS and spatial proteomics datasets (Fig. 4*E*). While Trpv1 was also included in the proteomic panel, we observed inconsistent staining across sections (data not shown) and thus could not confidently confirm that Trpv1 is a marker of a unique SMC subtype in the aortic root.

**Fig. 5:**
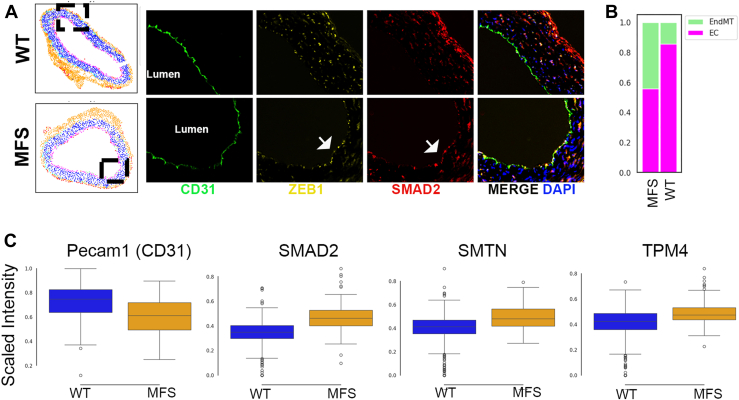
**Validation of endothelial-to-mesenchymal (EndMT) phenotype using spatial proteomics**. *A*, representative image showing designation of Zeb1 and Smad2 positive CD31-expressing ECs as indicative of EndoMT. Dot plot to the left shows assigned phenotype based on spatial proteomics analysis, with *magenta* cells representing “normal” ECs (?Zeb1-/Smad2-/CD31+) and *light green* cells representing EndMT phenotype (?Zeb1+/Smad2+/CD31+). *B*, Proportion of EndMT *versus* “normal” EC phenotype between MFS and WT. *C*, relative intensity of EC marker Pecam1, TGFB signaling and EndMT regulator Smad2, and the SMC/Mesenchymal markers Smtn and Tpm4 in “normal” ECs of WT and MFS genotype. EC, endothelial cell; MFS, Marfan’s syndrome; Pecam1, platelet endothelial cell adhesion molecule 1; SMC, smooth muscle cell; Smtn, smoothelin; TGFβ, transforming growth factor β; Tpm4, tropomyosin alpha-4 chain; Zeb1, zinc finger E-box-binding homeobox 1.

Another novel observation was the potential EndMT phenotype enriched among MFS ECs. Using CD31 staining to identify ECs and Zeb1 and SMAD2 as markers of potential EndMT (Fig. 5*A*), we confirmed an increase in EndMT-marker positive ECs in MFS relative to WT within the spatial proteomics data (Fig. 5*B*). Furthermore, also consistent with the SCP-MS data, when examined separately from the overt (Zeb1 positive) EndMT-classified cells, even the “normal” ECs of MFS aorta exhibited trends toward EndMT progression, with lower Pecam1 intensity and concomitantly elevated Smad2, Smtn, and tropomyosin alpha-4 chain (Tpm4) intensity relative to WT (Fig. 5*C*).

### Comparison of Singe-Cell RNA and Protein in Marfan Mice Uncovers Key Differences in Cell Type Assignment

**Fig. 6:**
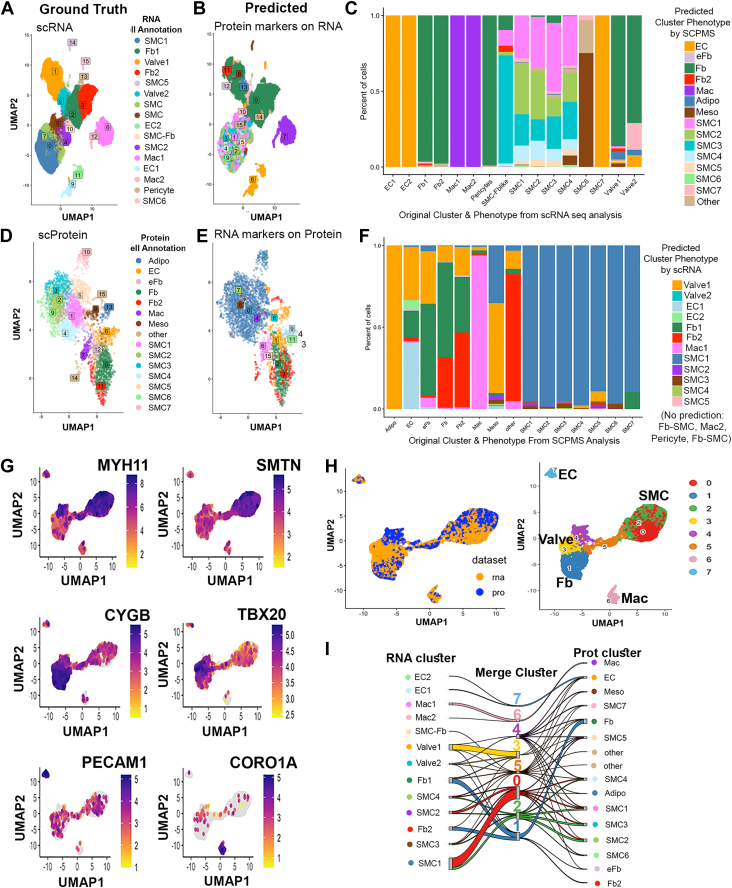
**Comparison of SCP-MS and scTranscriptomic sequencing data from male and female MFS and WT mice**. *A*–*F*, Seurat analysis of marker projections between transcriptomically defined and proteomically defined mouse aorta cell types. *A*, original cell assignments in proteomic data defined by proteomic markers. *B*, projection of RNA cluster phenotype assignment based on proteomic-markers. *C*, comparison of the proportion of cells assigned to a given predicted proteomic-defined cluster (*Bar* colors), according to the original RNA-defined cluster phenotype (*x* axis). *D*, RNA data defined by RNA markers. *E*, projection of RNA-defined cluster assignment onto proteomic data. *F*, comparison of the proportion of cells assigned to a given predicted RNA-defined cluster (*bar* colors), according to the original proteomic-defined cluster phenotype (*x*-axis). *G*, UMAP of multi-omic dataset displaying cell-type specific marker expression profiles for smooth muscle (MYH11 and SMTN), fibroblast (CYGB), valve (TBX20), endothelial (PECAM1), and macrophage (CORO1A) cells. *H*, UMAP of multiomic integrated datasets colored by dataset of origin (RNA *versus* proteomic, *left panel*), and Leiden-assigned clusters (*right*), with Leiden plot labeled for general cell type assignment based on marker expression patterns. *I*, Sankey plot demonstrating cell cluster assignment of a given cell from the transcriptomic (RNA clusters, *left*) or proteomic (protein clusters, *right*) datasets into their corresponding multiomic defined Leiden cluster from the LIGER integrated dataset (*middle*). MFS, Marfan’s syndrome; Myh11, myosin heavy chain 11; Pecam1, platelet endothelial cell adhesion molecule 1; SCP-MS, single-cell proteomics by mass spectrometry; scTranscriptomic, single cell transcriptomic; Smtn, smoothelin; UMAP, uniform manifold projection.

Previous studies have reported the single cell transcriptome of the *Fbn1*^*C1041G/+*^ mouse model used in this study, enabling us to explore the degree of similarity between the two single cell modalities. Using the same Scanpy pipeline applied to our SCP-MS results, we reanalyzed data from the Gene Expression Omnibus produced from single cell transcriptomic analysis of similarly aged *Fbn1*^*C1041G/+*^ and WT mice, generating a UMAP sorted plot of single cell RNA-seq derived cell clusters and marker genes that were comparable to those produced by the original authors Pedroza *et*. *al*. (Supplemental Fig. S13) (27). Using Seurat anchor marker analysis, we compared cell marker annotation between our proteomics and the previously published Pedroza *et*. *al*. transcriptomic datasets (37). In this analysis, trans-analyte plots in UMAP space were generated from cell type markers from RNA-seq to define cell types in the proteomic data and vice versa (Fig. 6, *A*, *B*, *D* and *E*, originally defined subtypes on left panels, projected trans-analyte defined cell types on right). To further visualize whether assigned phenotypes from one data modality transferred well to the other, we examined the proportion of a predicted phenotype assigned to the cells according to their originally designated phenotype (Fig. 6, *C* and *F*). While major subtypes were consistent in the trans-analyte analysis (*e*.*g*.*,* SMCs, fibroblasts, macrophages, and ECs), cell subtypes, such as different categories of SMCs, were less preserved. To represent this observation quantitatively, we computed adjusted rand index (ARI) scores comparing the original cluster assignment of cells to their predicted cluster assignments were they to be merged into the corresponding opposite dataset (*i*.*e*.*,* cell phenotype as assigned by SCP-MS markers, *versus* cell phenotype as assigned by the RNA-analysis based markers, prediction outputs provided in Supplemental Tables S11 and S12). When we attempted to reclassify proteomically defined cell types using transcriptionally defined cluster markers, the ARI comparing the original to predicted cluster assignments was 0.25. Conversely, when reclassifying transcriptionally defined cells using proteomically identified markers, the ARI comparing the original to predicted cluster assignments was 0.18. By contrast, when we subset out just the SMC subtypes from both SCP-MS and RNAseq datasets, the corresponding ARIs were both 0.01, reflecting essentially zero alignment between protein and RNA classified SMC subtypes.

To further explore similarities and differences between transcript and protein based single-cell data interpretation, we then performed an integrated multi-omic analysis using the tool LIGER, which combined the RNA and protein datasets into a single data matrix. Reclustering using leiden (Fig. 6*B*) with assigned cell types based on composite marker expression generated a new plot in UMAP space (Fig. 6*C*). The expression level and location of several of these composite cell type markers demonstrate the specificity of these genes for defining the multi-omic clusters (Fig. 6*D*). A Sankey plot was used to illustrate the contribution of each cluster from the RNA dataset and the protein dataset to the merged Leiden clusters from the multi-omic LIGER algorithm (Fig. 6*E*). Tracing the path of transcript-defined or protein-defined cell types into multiomic-defined cell-type clusters, it is evident that even with multi-omic integration, the two datasets are only broadly matched by general cell types, wherein more subtle cell subphenotypes did not contribute in any clear patterns to the integrated clusters within the LIGER dataset.

### Multi-omic Analysis of SMCs Uncovers Unique Gene Signatures Enriched in Marfan Mice

**Fig. 7:**
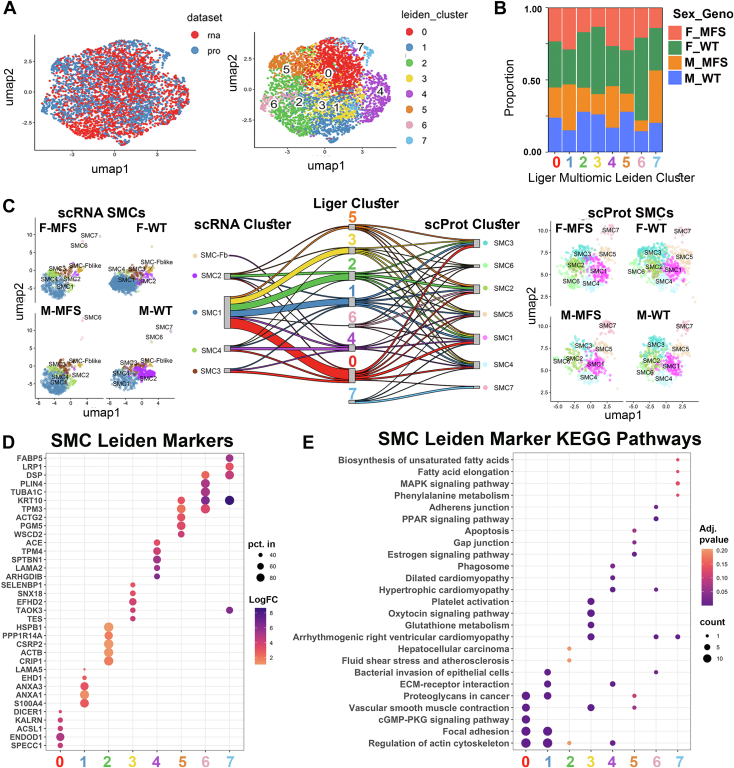
**Focused multiomic analysis of smooth muscle cell (SMC) subtypes**. *A*, UMAP projections of SMCs as defined within respective RNA and proteomic datasets, demonstrating integrated mixing (*left panel*) and leiden SMC subtype clustering (*right panel*). *B*, proportion of multi-omic SMCs by mouse sex and gender separated across leiden defined SMC subtypes. *C*, Sankey plot demonstrating mixing of cells from originating RNA-defined (*left*) and proteomic defined (*right*) clusters into multi-omic defined (*middle*) clusters. *D*, dot plot depicting the top shared marker features from the RNA and protein datasets for each LIGER derived Leiden cluster. *E*, dot plot describing significantly enriched KEGG pathways from the shared marker features for each LIGER derived Leiden cluster. KEGG, Kyoto Encyclopedia of Genes and Genomes; SMC, smooth muscle cell; UMAP, uniform manifold projection.

To further examine whether similarities in cellular subphenotypes could be identified from the multi-omic analysis, we focused specifically on SMCs. To do this, we subset each dataset to only the cells annotated as SMC based on their dataset-of-origin classifications done with canonical marker expression, resulting in 2200 total SMCs from the RNA dataset and 2031 total SMCs from the protein dataset. These two datasets were integrated using LIGER, and a SMC-specific plot of cells in dimension reduced space derived from UMAP was generated (Fig. 7*A* left), with seven unique SMC subtype Leiden clusters estimated (Fig. 7*A* right). The proportional contribution of cells based on sex and genotype to each Leiden cluster demonstrates significant enrichment in cluster 6 among mice known to typically have smaller aortic dimensions (7.5% in WT female, 4.1% in MFS female, 1.5% in MFS male, and 2.7% in WT male) and enrichment in clusters 1 and 4 in the MFS mice (22.8% *versus* 12.5% in MFS *versus* WT female and 26.3% *versus* 12% in MFS *versus* WT male for Cluster 1; 12.6% *versus* 8.7% in MFS *versus* WT female and 14.4% *versus* 8.0% in MFS *versus* WT male for Cluster 4) (Fig. 7*B*, Supplemental Fig. S14, and Supplemental Table S13). Sankey plot demonstrated relatively poor overlap between RNA-defined SMC subtypes and protein-defined SMC subtypes relative to how they contribute to the integrated clusters (Fig. 7*C*). To understand the biological similarities and differences between these multi-omic-defined clusters, we determined the top marker features (*e*.*g*.*,* shared RNA transcript and protein between datasets) for each cluster (relative to all other clusters) (Fig. 7*D* and Supplemental Table S14), and performed a KEGG pathway enrichment analysis (Fig. 7*E* and Supplemental Table S15). Unsurprisingly given that all cells used in this analysis were defined as SMC based on canonical SMC marker abundance, the SMC markers did not meet the cutoff of “top marker” for any of the clusters (*e*.*g*.*,* none of these key canonical SMC markers were uniquely intense or only expressed by a single SMC subtype). That being said, there were noticeable differences between clusters in SMC marker intensities, with the MFS enriched clusters 4 and 7 demonstrating lowest combined levels of most contractile markers (Supplemental Fig. S15). In terms of top markers, two particularly interesting markers of MFS enriched cluster 4 were Tpm4 and angiotensin-converting enzyme (Ace), markers of MFS enriched cluster 1 included S100A4 and Annexin A1 and A3, and cluster 7, which trended toward upregulated in MFS and was solely proteomically defined, showed markedly high Lrp1 among its top five differential features. KEGG pathway analysis of expanded marker sets (up to 100 top significant markers with Log_2_FC > 1) revealed that clusters 0, 5, and the WT enriched cluster 3 were particularly enriched in smooth muscle contraction features, while cluster 1 and 4 showed enrichment for actin cytoskeletal organization and ECM-receptor interaction, with cluster 4 also uniquely enriched in features related to the phagosome.

### Validation of Multiomic Phenotype Markers in SMCs Using Spatial Proteomics

**Fig. 8:**
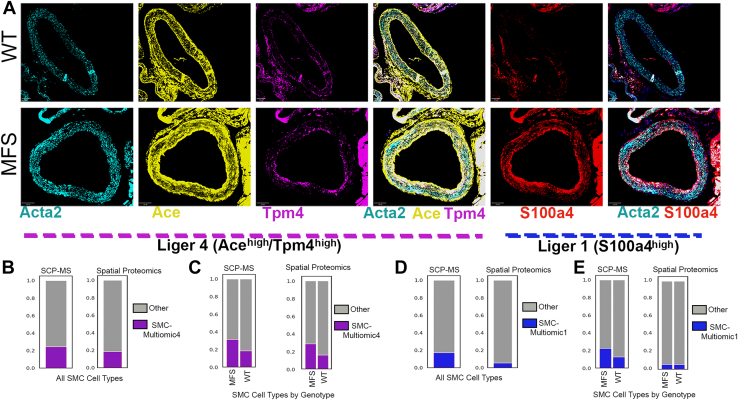
**Spatial proteomic validation of multiomic SMC Liger clusters 1 and 4**. *A*, expression of multiomic-defined markers of LIGER cluster 4 (Ace and Tpm4) and LIGER cluster 1 (S100a4) with overlay against Acta2 for smooth muscle cell visualization. *B*, relative proportions of SMC Liger cluster 4 as quantified between the SCP-MS and spatial proteomics datasets. Data are shown as proportion of LIGER-cluster 4 (*purple*) relative to all other SMCs (*gray*). *C*, relative proportions of SMC Liger cluster 4 split by genotype and compared between the SCP-MS and spatial proteomics datasets, data are shown as proportion of LIGER-cluster 4 (*purple*) relative to all other SMCs (*gray*) of a given genotype. *D*, relative proportion of SMC Liger cluster 1 cells relative to all other SMCs measured in the SCP-MS or spatial proteomics datasets, data are shown as proportion of LIGER-cluster 1 (*blue*) relative to all other SMCs (*gray*). *E*, relative proportions of SMC Liger cluster 1 broken out by genotype and compared across the SCP-MS and spatial proteomics datasets, data are shown as proportion of LIGER-Cluster 1 (*blue*) relative to all other SMCs (*gray*) of a given genotype. Ace, angiotensin-converting enzyme; SCP-MS, single-cell proteomics by mass spectrometry; SMC, smooth muscle cell; Tpm4, tropomyosin alpha-4 chain.

To confirm the multiomically defined SMC subtypes, we included antibodies for S100a4 as a marker of the MFS enriched multiomically defined subtype 1 and both Ace and Tpm4 as markers of the MFS enriched multiomically defined subtype 4 (Fig. 8). In both SCP-MS and Spatial Proteomics dataset, approximately 20% of all SMCs were Ace^high^/Tpm4^high^, corresponding to multiomic-cluster 4 designation. Also consistent with the multiomic-analysis, there was a higher proportion of Ace^high^/Tpm4^high^ SMCs in MFS relative to WT aortas. When examined as a stand-alone marker, we also confirmed very similar proportions of S100a4+ SMCs between SCP-MS and spatial proteomics experiments (Supplemental Fig. S19); however, when considered as a unique SMC subtype marker, very few SMCs were uniquely S100a4^high^ in the spatial proteomic dataset (*i*.*e*.*,* not also Ace^high^ and/or Tpm4^high^, Fig. 8*C*), with no difference in purely S100a4^high^ SMCs between genotype (Fig. 8*D*) suggesting that at least at a protein level, the multiomically defined clusters 1 and 4 may not be distinct.

## Discussion

This manuscript describes an early example of the application of SCP by mass spectrometry to a heterogenous mammalian tissue, using it to address a complex biological question. Mouse vasculature was chosen as a model tissue due to its assortment of well-defined cell types. Marfan aortic root aneurysms were compared to normal mice as a pathological variation as aneurysm is known for its shift in subtypes of SMCs and has been previously assayed through single-cell RNA sequencing, providing us with an ability to directly compare the two single cell modalities head to head and combine them into one master signature (27). Male and female single-cell proteomes were also compared given the well-characterized sex-differences in aneurysm severity in both mouse models and human patients with aneurysm (38).

Mouse aortic root tissue is extremely small starting material, yet we successfully profiled 3475 cells with at least 200 proteins across 12 biological replicates. These cells clustered using the Leiden algorithm into 16 groups featuring protein markers consistent with all known major cell types in aortic mouse tissue. This included several subtypes of abundant cells like SMCs and fibroblasts. On average, we were able to detect roughly 500 proteins per cell which represents about 15% of the proteome detected using bulk tissue proteomics on similar samples (39). This number of proteins per cell is consistent with our own previous reports (10), but lower than recently released preprint results on dissociated mouse tissue (40) and human pancreatic islet cells (41). We attribute the lower numbers in our report to the fact that our experiments were completed on the first-generation Bruker TIMSTOF SCP, which has now undergone several iterative advancements in sensitivity alongside the introduction of the Thermo Fisher Orbitrap Astral which is also capable of single-cell level sensitivity. Therefore, the lower yield is likely due to the application of earlier technology. Indeed, protein quantification numbers here are far more comparable to those done on similar LC MS set ups from “generation one” instrumentation (9). Thus, this report presents a “snapshot” of the capabilities of SCP relative to the instrument sensitivity available at the time of data acquisition (*e*.*g*.*,* Oct-Dec 2023). Use of the nanoDTSC approach enabled direct single cell analysis with reasonable throughput and avoids potential challenges to detecting rare cell types in heterogenous mixtures that may be present when multiplexing is used to boost peptide detectability. However, to gain sufficient throughput, nanoDTSC gradients are relatively short, and this may also limit ultimate proteomic depth in our results relative to other reports using longer gradient lengths. Indeed, even with the 15-min analytical time of the nanoDTSC approach here, the acquisition of nearly 5000 cells consumed nearly 3 months of instrument time. Additional efficiencies in both gradient length, multiplexing options, and more sensitive MS instruments will further advance ongoing SCP-MS experiments that aim to capture the full heterogeneity of complex biological systems. Proteomic depth notwithstanding, we made numerous biological observations, some of which corroborate existing knowledge which supports the validity of our data, but more importantly, we made a handful of novel observations leading to new hypotheses regarding the role of Fbn1 in aneurysm pathogenesis.

For instance, SMCs were by far the most abundant and heterogenous cell type, as anticipated given the histological structure of the aorta and the relative pluripotency of SMCs. Informed by expression levels of canonical SMC marker proteins like Myh11, Smtn, Vcl, and Tagln, we confirmed that these data recapitulate well-described SMC states, which vary on a continuum of highly contractile to modified, proliferative and/or secretory phenotypes (42). One novel observation from SCP-MS results was the identification of new markers representing the “modified” SMC states (Prss2, Lrp1, and Trpv1), two of which (Prss2 and Lrp1) were validated by spatial proteomics. Lrp1 is a well characterized SMC receptor that plays a critical role in the integrity of vessel walls. Disruptions in the Lrp1 gene have been linked to both abdominal and thoracic aneurysms (43, 44), The upregulation of Lrp1 in modified SMCs was therefore surprising, given the tendency for these modified cells to be more abundant in aneurysmal tissue and the purported protective role of Lrp1 in aneurysm. We hypothesize that upregulation of Lrp1 may therefore be a compensatory response. Interestingly, three of the four modified clusters expressing higher Lrp1 also expressed elevated Prss2. Prss2 has not previously been described in SMCs, and we thus validated its expression in SMCs using spatial proteomics, showing nearly identical proportions of Prss2+ SMCs between SCP-MS and spatial proteomic experiments on an independent set of mice, and also corroborating elevated Prss2+ cell proportions in MFS. An interesting report in pancreatic cancer indicates that Prss2 can act as a ligand for Lrp1 in tumor cells, ultimately working together to promote cell proliferation and malignancy (45). Two of the three clusters co-expressing higher levels of Prss2 and Lrp1 (SMC1, SMC7) had notably higher proportions in MFS *versus* WT aorta. Future studies to explore whether this interaction in SMC is pathogenic or compensatory to aneurysm progression in MFS are warranted, and represent one new hypothesis generated from the current body of work.

One of the principal objectives of this study was to identify novel cell specific differences between WT and Marfan mice and/or according to mouse sex. Testing for DEPs within each cell cluster type, comparing either sex or genotype difference, generated relatively few differences that met FDR threshold. Loosening of statistical stringency to set significance relative to nominal *p* values increased possible DEP detection. We believe that this observation reflects the relative stringency of our statistical approach. Many studies use individual cells as “replicates” in these comparative statistical tests, which has been criticized for artificially inflating statistical power (amplifying type 1 error). Collapsing protein intensities into a single observation per biological sample (the pseudobulk approach) addresses this concern but may artificially constrain statistical power (amplify type 2 error). To balance these two opposing risks, we chose the linear mixed-effects model, which leverages multiple testing within a biological replicate while still constraining type 1 error risk. That being said, our power analysis indicates that the relatively small sample sizes in each of our biological groups or lower sampling in terms of number of cells within a given cluster of a given biological group still contributes to a general lack of power to detect more than the very largest DEPs within cell types. Alternative comparisons, such as pathway-based enrichment analysis (46), are one other option to work around challenges of low power and could be considered for future efforts with this or other similar datasets. Increasing the number of cells sampled per replicate in future studies will also improve statistical power when the same approach is used. Despite relatively low numbers of stringently defined DEPs, some interesting observations still emerged. Fibroblasts showed the largest number of intracell type DEPs between males and females. This intriguing finding is consistent with recent reports on key sex differences in fibroblasts of the heart, and identifies this cell type as a potential key mediator of sex-differences in vascular risk and resiliency in aneurysm, a possibility that warrants more extensive exploration in future studies.

The comparison of DEPs by genotype across cells highlighted a pattern of protein expression in ECs consistent with increased EndMT in MFS aorta, an observation that was corroborated using external RNAseq data from an independent study as well as our spatial proteomics analysis on separate MFS and WT mice. EndMT has been demonstrated as a correlate of aneurysm pathogenesis in patients with bicuspid aortic valves (47). A recent conflicting study concluded that EndMT likely plays a more significant role in Marfan aortopathy compared to other forms of thoracic aneurysms and implicate transforming growth factor β (TGFβ) signaling as at least one mechanistic factor driving this process, though ages were significantly different between groups, with MFS patients being nearly 2x younger on average than comparison groups, confounding whether increased EndMT was age or disease related (47, 48). Another study detected similar reductions in EC “marker” proteins like Pecam1, VE Cadherin, and endothelial nitric oxide synthase in induced pluripotent cell derived ECs from MFS *versus* normal patients, further corroborating our results indicating abnormal ECs in MFS (49). Taken together with the findings reported here, we identify some of the first evidence for the existence of elevated EndMT in MFS aorta, setting up a strong justification for future studies to clarify the role this phenotypic shift of ECs has in aneurysm pathogenesis.

It is well-known that transcript and protein expression levels do not often correlate strongly, including when measured at the single cell level (9, 50). We sought to understand how this lack of consistency may affect the inferences made and conclusions drawn regarding cell phenotypes that may be present in a given biological system and how those phenotypes might change in disease states. Both trans-analyte and integrated analyses revealed that outside the overlap in major cell type identity, there was very little similarity between the two omic modalities in describing the heterogeneity of cell types of the aorta (*e*.*g*.*,* SMC subphenotypes). These differences could be entirely due to technical explanations including differences in the genetic background strain of the *Fbn1*^*C1041G*^ mutants (C57Black6 for scTranscriptome and 129 for SCP-MS); the relatively large number of RNA features (1000s) compared to protein features (100s) causing poor feature overlap; and different sources of technical and biological variation across studies that were unaccounted for in our models; the computational approach for integration; and the mismatch in biological replicates between the two datasets nevertheless, as the disagreements between modalities were not subtle, particularly in how SMC subtypes were described, our results suggest that proteomic and transcriptomic descriptions of single-cells could lead to differing conclusions regarding underlying biology, an intriguing hypothesis that warrants further study using direct comparisons made from complex heterogenous datasets.

The LIGER integrated SMC projection identified a series of unique clusters of multi-omic-defined cells, some of which demonstrated altered proportions across WT and Marfan mice. Key functional components of the contractile apparatus were enriched in multi-omic clusters with higher contributions from WT cells, which highlights the importance of contractility and its regulation in maintaining vessel wall integrity. Separately, integrated Leiden clusters with significant contributions from Marfan cells contained genes related to angiotensin, TGFβ, and protease signaling, all well established players in MFS aneurysm biology. Thus, the multiomic analysis generated unique, complementary cell phenotype classifications that reflect the combined influence of both the transcript and protein gene product levels in a given cell. Among the clusters, we found the multiomic cluster 4, marked by high Ace and Tpm4 expression, to be upregulated in MFS which was further corroborated in our spatial proteomic validation experiment. Although traditionally considered an endothelial-enriched protein, expression of Ace specifically on medial SMCs has recently been mechanistically linked to atherosclerotic propensity in mouse models, suggesting an important role for this protein in SMC biology in MFS and introducing another novel hypothesis generated by this study for application to aneurysm pathogenesis (51). Also of interest, Tpm4 has been highlighted as upregulated during SMC dedifferentiation and is enriched in atherosclerotic plaque SMCs (52). Taken together, from a multi-omic analysis, it is clear that cluster 4 is transcribing, translating, and stably expressing higher levels of markers for SMC de-differentiation. These markers have been linked previously to other aortic pathologies, but our data are the first to implicate them in MFS aneurysms. Targeting these genes could represent novel pathways to prevent phenotype transitions in Marfan SMCs, a process that seems the key to developing new therapeutic applications.

In addition to slightly lower average protein identifications per cell, there are some other limitations or considerations in this study. Preprocessing and informatics strategies for SCPMS are in early stage and active development and there is not yet clear consensus on ideal approaches for given experimental preparations and biological questions. Here, we chose a normalization, scaling, and batch correction approach informed predominantly by performance in single-cell transcriptomics workflows. Recently, combat was identified as one of the three “best” performing batch correction methods for SCP analysis (25), lending some confidence to our selection. That being said, differences in normalization and batch correction can substantially change downstream dimensionality reduction, clustering, and biological interpretation and thus these data should be interpreted with appropriate caution. Our ability to validate key biological findings using spatial proteomics lends some support to the validity of our choices in methodology. In addition, contrary to scRNA sequencing studies, there is not an agreed upon strategy to assess cell quality and cell stress in the SCP-MS results. In the current study, the potential contaminant plasma proteins Alb, Hbb1, and Hbb2 were examined across processing batches to determine if blood-related proteins bound to the individual cells contributed to the final proteomic results. We also compiled a “HSP score” as a possible index of cell stress; however, it is unclear whether the <4 h end-to-end processing of the aorta to lysed and frozen cells would be sufficient to introduce measureable levels of increased stress-related protein translation and abundance. There is a need to further consider and expand upon these indicators as a means to possibly filter nonrepresentative or otherwise undesirable cells from the dataset in preprocessing. We did use a viability dye in order to ensure that only live cells were sorted into wells and lysed, which is at least a high level indicator that the cells were in a reasonable state ahead of their proteomic data acquisition. Another consideration is that the scRNA sequencing data did not come from cells of the same animals, or even the same colony, as the SCP-MS data. Therefore, it may be that datasets isolated from the same tissues or even the same cells will yield improved alignment relative to that seen here.

In summary, this SCP-MS analysis has revealed novel insights into MFS specific cellular phenotypes, including the potential involvement of endMT in altered endothelial function in MFS, as well as likely key roles for SMC Ace, Tpm4, Lrp1, and Prss2. Through comparisons to transcriptome-defined single cells and integrated multiomic analysis, we show that there is complementary information gleaned from SCP-MS analysis and its integration with transcript data. With emerging reports of dual-omic extraction of protein and RNA from the same individual cells now applied to simplified *in vitro* systems (50), our findings reported here support the importance of pushing such approaches to be robust in complex biological tissues.

## Data Availability

Raw data (including library generation files), processed counts matrices, and the Scanpy and Seurat files used to generate these results are deposited on the MassIVE server for public access. Identifier: MSV000097486 (Reviewer login: MSV000097486_reviewer, Password: JVE12345!), a description to aid in navigating the data share environment is provided in Supplemental Table S16.

## Supplemental Data

This article contains supplemental data (18,19,53).

## Conflict of Interest

The authors declare no competing interests.
