# Supplementary material for: Single-Cell Proteomics Reveals Novel Cell Phenotypes in Marfan Mouse Aneurysm
Source: Mol Cell Proteomics. 2026 Mar 3;25(4):101549. doi: 10.1016/j.mcpro.2026.101549 (PMC13090654; doi:10.1016/j.mcpro.2026.101549)
Supplement: Supplemental — Figures [file mmc2.docx]

*Supplemental Methods*

**Preparation of Single-Cell Suspensions from Mouse Aortic Root.** Mouse work was performed under the approved Cedars-Sinai IACUC protocol #iacuc008535. Mice were sacrificed between 12-13 weeks of age using high concentration isoflurane inhalation. The inferior vena cava of the mouse was cut to let blood flow out and 10 ml of ice-cold PBS was injected into the left ventricle. Aortic roots from the aortic valve to 1-2mm above the sinotubular junction were dissected and cleaned of most valvular, myocardial, other connective tissue and excess/loosely adhering pericardial fat. Aortic roots from two mice of matching genotype and sex were rinsed of blood with ice-cold PBS and then transferred into prewarmed enzymatic digestion cocktail prepared with 300U/ml of collagenase Type II (Worthington CLS-2), and 3 U/ml of elastase (Worthington ES) in Hanks’ balanced salt solution (HBSS). Aortas were diced into small pieces and incubated at 37C for 40 min to allow dissociation of cells from the connective extracellular matrix. The digestion solution was gently passed 10x through a 21-gauge needle to further liberate remaining cells and enzymatic reaction was stopped with 20% fetal bovine serum (FBS) in Dulbecco’s Modified Eagle Medium (DMEM). The mixture was then passed over a 70um cellular strainer to collect dissociated cells and remove matrix tissue and cell clumps. The strainer/filter was washed with ice-cold PBS and cells suspension was centrifuge at 500g at 4C for 5min. Cell pellets were resuspended in 200ul ice-cold PBS. Cell suspensions were then labeled with Sytox Green nuclei acid stain. Specifically, 1ul of 5mM Sytox green solution was added to the 500uL cell suspension (0.5x10^6^cells/mL) and incubated at room temperature for 15 minutes.

**Direct Single-Cell Liquid Chromatography Separation and Mass Spectrometry Acquisition.** The contents of each well on the 384-well plate were resuspended in 20uL of 0.1% formic acid 2% acetonitrile using the autosampler and injected onto one trapping column (Exp2 170nL packed with 10um diameter PLRP beads, Optimize Technologies). Concurrently the previous sample trapped on an identical trapping column was eluted onto the analytical column (15cm x 75um filled with 1.9um particles PepSep Bruker) and separated using a binary reversed phase gradient (0.1% formic acid in water to 0.1% formic acid in 80% acetonitrile 20% methanol) at 500nL/min flowrate. The gradient was delivered as follows: 9%B to 25%B over 8 minutes 25%B to 38%B over 4.6 min, followed by a 98%B wash at 98%B at 1000nL/min for 1.2 min and equilibration to 9%B at 100nL/min for 1 min (15 min total run time or 96 samples/day). The separated peptides were sprayed through a 20um ZDV emitter installed in the Bruker captive source at 1700V with the dry gas set at 3.0L/min and 200C glass capillary temperature. Data were acquired by data-independent acquisition parallel accumulation-serial fragmentation (DIA-PASEF) with the ion accumulation and trapped ion mobility ramp set to 166 ms. Each MS1 scan was followed by 90m/z wide DIA scans spanning 300–1200m/z and 0.6–1.43 1/K0 with 4 trapped ion mobility ramps (0.86s total cycle time).

**Generation of the Sample-Specific Peptide Library.**

*Bulk DDA library.* Magnetic assisted cell sorted (MACS) CD31+ and CD31- fractions of dissociated mouse aortic root cells as well as MACS sorted CD45+ peripheral blood mononuclear cells, all collected from a WT and MFS mouse, were run separately by cell source / genotype (e.g., N=6 final DDA samples run). Isolated cell pellets were lysed using 8M Urea/5% SDS and processed for tryptic digestion using standard procedures except that no reduction of cysteine residues (e.g., no DTT or IAA alkylation step) was performed in order to produce peptides consistent with the single cell sample processing protocol. Protein lysates were processed using the STRAP manufacturer recommended protocol (Protifi, Fairport, NY). Peptides were analyzed on the Bruker TIMSTOF SCP instrument using the same LC configuration but with data dependent acquisition (PASEF-DDA). Both accumulation and ramp times were set to 166 ms with DDA scans acquired over 5 ramps within a polygon spanning 240-1700m/z and 0.6-1.5 1/K0 for a cycle time of 1.03 s. The resulting raw files were searched against the uniprot mouse (species UP000000589) reviewed, canonical protein database downloaded March 2024 with 17,314 entries (including the CrAP-ome common contaminant sequences) using the FragPipe MS Fragger workflow^1^ (version 21.1; Fragger version 4.0), with filtering to 1% peptide and protein FDR using Philosopher (version 5.1.0) and integrated into a ezPQP (version 0.1.42) library (the full parameter file from this search is provided in the MassIVE data share). For the search, precursor and fragment mass tolerance was set to 20ppm, up to two miss cleavages were allowed, variable modifications included oxidized methionine (M + 15.9149) and N-terminal acetylation (^ + 42.,0106). As no reduction step is used in the digestion process, there were no fixed modifications set.

*LibraryFree DIANN selected single cell.* The full dataset of single cell files were initially searched against our previously published preliminary library using DIANN v1.8.1^2^ with precursor and fragment mass tolerance set at 15 PPM, FDR set to 1%, no normalization used and high precision IJMS quantification using MaxLFQ for protein-level inference. The resulting protein matrix was analyzed using the Single Cell Analysis in Python (Scanpy)^3^ workflow, as described below, and preliminary cell type assignments were made according to leiden clustering and canonical cell type markers. From these preliminary cell type assignments, the single cell data files with the top 10 highest protein identification counts from each cell category (SMC1/2/3 representing three SMC subtypes, Fibrobast, Macrophage, Adipocyte, Epithelial/Mesothelial and Endothelial) were subsequently run in DIANN version 1.8.1 using a library free search. Search settings were as follows: Uniprot Mouse canonical and reviewed database (August 2022) with 17,125 entries; up to two missed cleavages; both precursor and fragment mass tolerance at 15 PPM, no fixed or variable modifications, false discovery rate cut off set to 1%, no normalization, and high precision IJMS quantification using MaxLFQ for protein-level inference. The product of the library free search includes a spectral library with predicted RT and measured ion mobility. These sample-specific spectral libraries from each searched cell type category (SMCs, endothelial cells, adipocytes, fibroblasts, macrophages/immune cells, mesothelial/epithelial cells) were sequentially merged, by appending only unique peptides not seen in any of the other libraries.

*Final Merged Library.* The final library was produced using a sequential merge in which first only peptides unique to the library free search were appended to the Bulk DDA library. Subsequently, only unique peptides from the preliminary library were added to the previously merged library of Bulk DDA and Library Free search results. The final library contained 45,287 peptide precursors and 5,345 protein groups and, given the merge strategy, had a max peptide FDR of 10% (summed 1% FDR from each contributing library) and was used for the final single cell analysis. Methods for each are summarized in **Supplementary Figure 1 A-E.**

**Prediction of leiden cluster assignments between scRNA and SCPMS datasets.**

To determine how well a cell cluster phenotype from one dataset could be predicted by the cluster information from the other dataset, we first used two tools within the Seurat package, ‘FindTransferAnchors’ and ‘TransferData’. First, using a combined PCA cell ‘anchor’ pairs (one from each respective dataset) are identified within the same ‘neighborhoods’, filtered for quality / confidence and assigned an anchor score. Additional details can be found on the Satija lab website (<https://satijalab.org/seurat/reference/findtransferanchors>). Next, using the ‘TransferData’ function (<https://satijalab.org/seurat/reference/transferdata>) these anchor scores are used to predict the categorical leiden assignment from the reference dataset most likely to apply to a cell from the prediction dataset. For example, a cell assigned leiden cluster 1 (SMC1) in the SCPMS would be predicted to be leiden cluster 1 or some other leiden cluster in the scRNA dataset? From this analysis, we can project the predicted leiden cluster matching to the cell types assigned in one dataset onto the other analyte type’s UMAP, examining whether cells that cluster together in that dataset also match to the same leiden cluster assignments predicted by the reference dataset. When this occurs, we interpret this to indicate strong overlap in latent molecular definitions of the cell type clusters between the two datasets. Summarized in the figure, above.

**‘FindTransferAnchors’**

- PCA on combined data
- Identify pairs from either Protein or RNA that share a ‘neighborhood’
- Filter low confidence anchors
- Assign anchor scores

**‘TransferData’**

- Pass categorical Leiden assignment informed by RNA leiden to cells in the Protein (or vice versa) based on Anchor scores


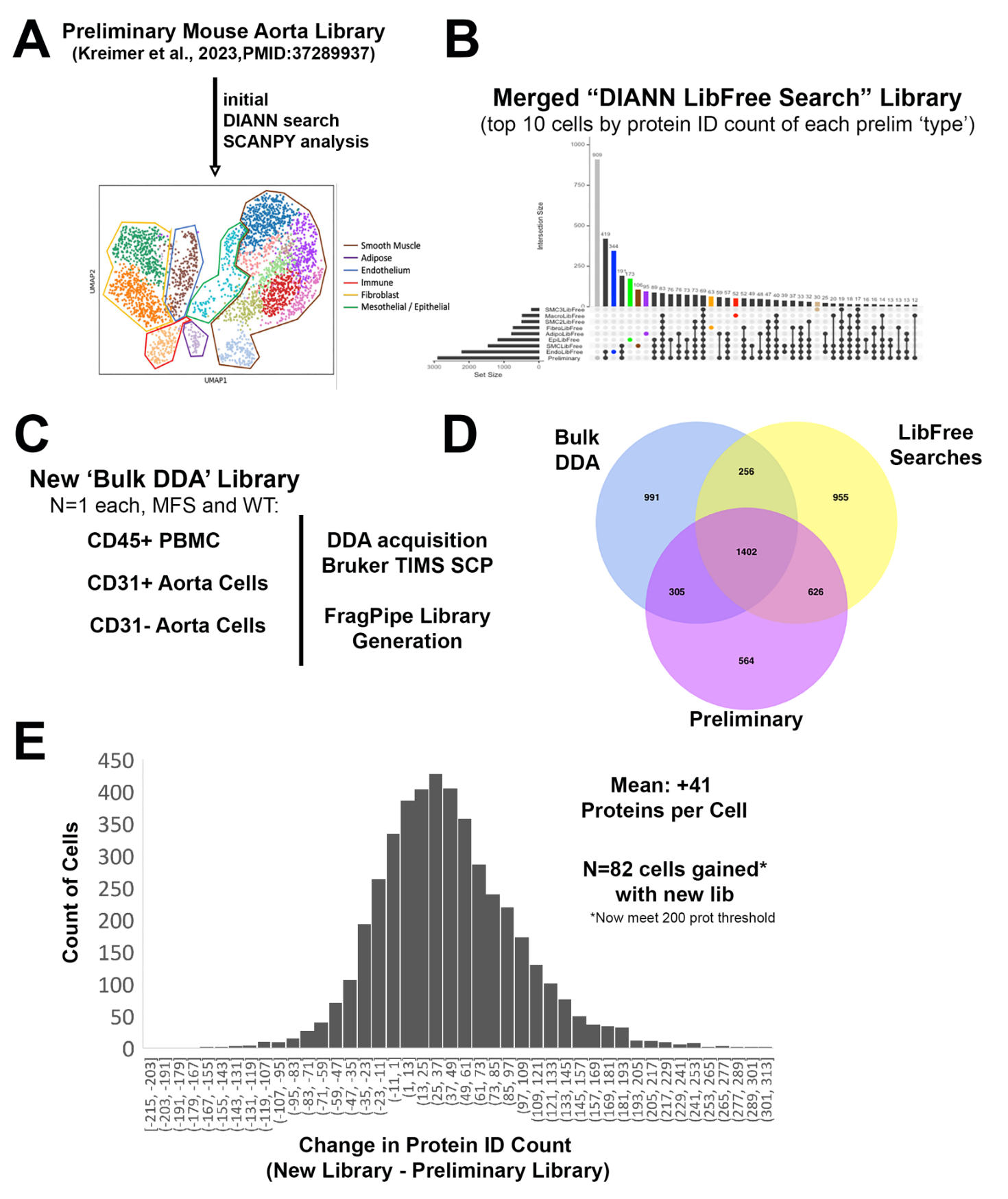
**Supplementary Figure 1.** **Assembly of the sample specific peptide assay library used for single cell peptide and protein identification and quantification**. (A) Initial clusters and cell IDs from search against published, preliminary library. (B) Overlap in results between cell-type guided, library free searches yields >1000 proteins not in original library. These results are assembled into the ‘LibFree Searches’ library. (C) Schematic for a newly acquired sample specific library from N=1 Marfan’s syndrome (MFS) and N=1 wild-type (WT) mouse. (D) Shared and unique proteins from each of the three unique libraries eventually assembled into the final, merged library. (E) Distribution of change in proteins identified per cell in the search against the new vs old library.

**
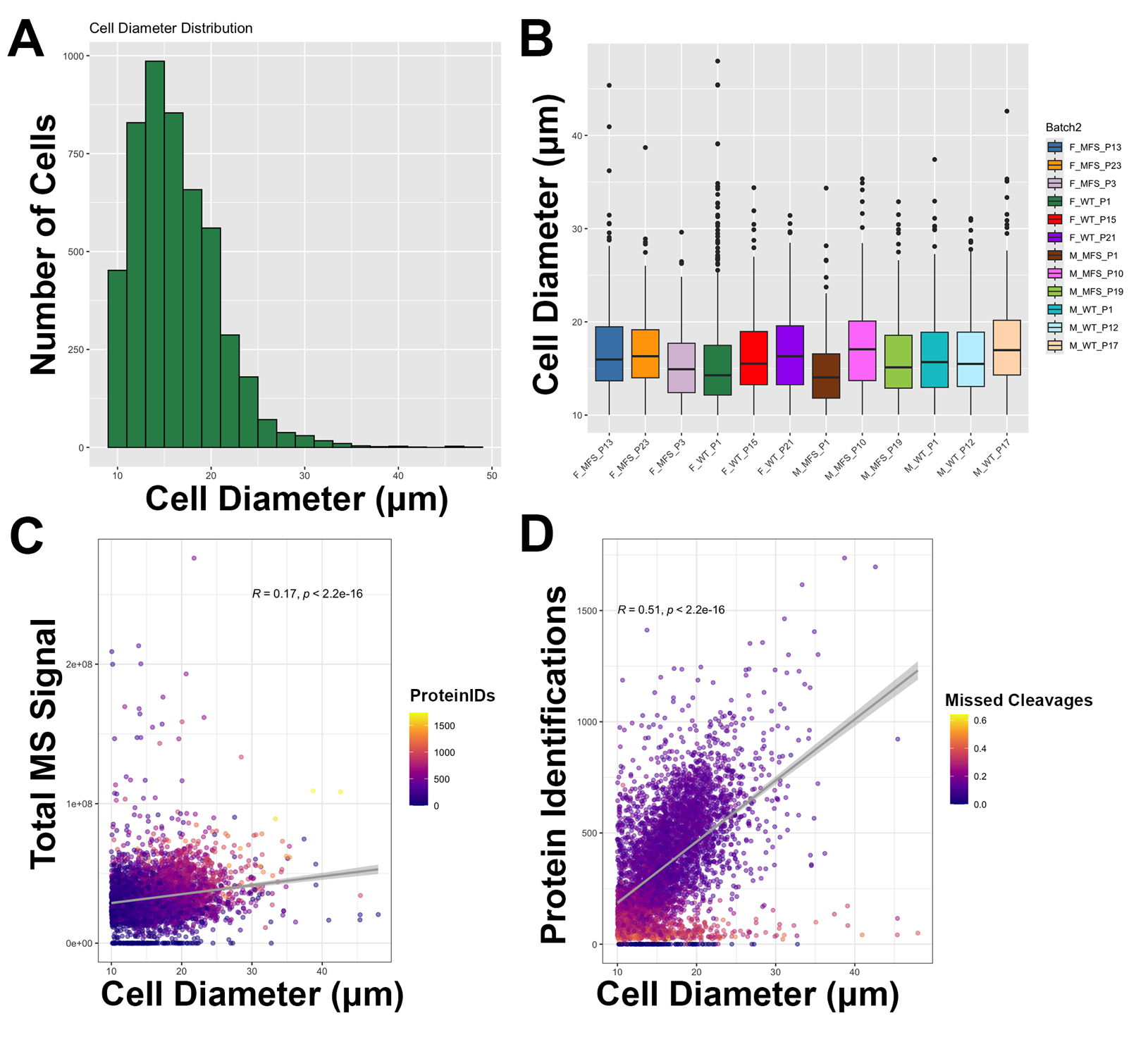
Supplementary Figure 2**. Distribution of cell diameters dispensed by the CellenONE sorter across the entire experiment (A) and as boxplots of cell diameters dispensed within each plate batch (B). After completing LC MS the relationship between cell diameter and total MS signal (C) and total protein group identifications (D) were plotted with dots coloed according to number of protein IDs or DIANN estimated missed tryptic cleavages, respectively.

**
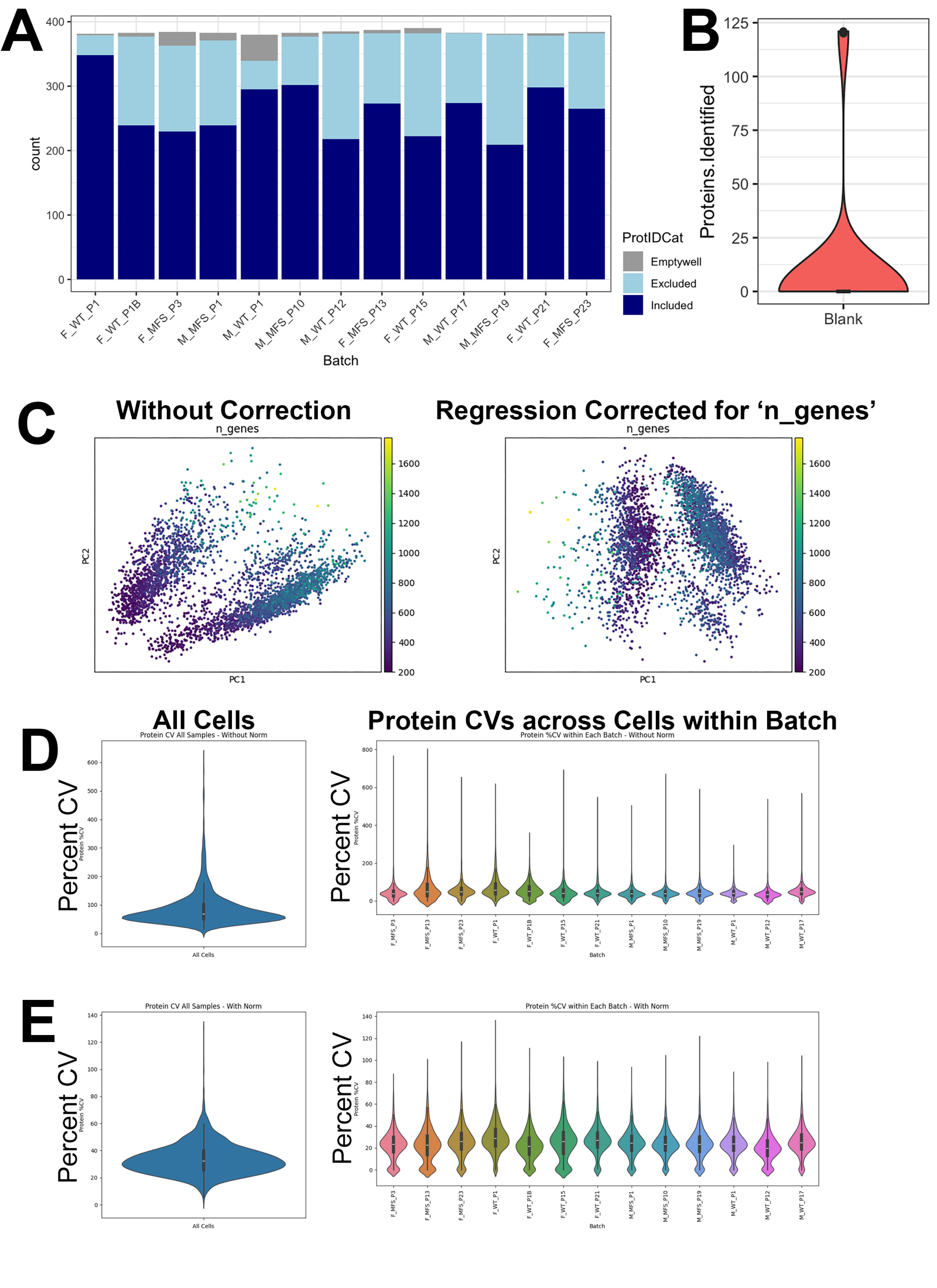
Supplementary Figure 3.** Summary of MS search outcomes for all cells run by LC MS for each plate (A), categorized as ‘included’ if >200 proteins were identified, ‘excluded’ if <200 (but more than zero) proteins were identified, and ‘empty wells’ if there were no proteins identified in the LC MS run of a given well. (B) Searching of blank runs against the same library yielded mostly zero protein identifications. (C) Visualization of the number of protein IDs per cell (n_genes) as distributed along principal components (PCs) 1 and 2 before (left) and after (right) applying a regression function to minimize the effect of protein identification depth on cell-to-cell proteomic variance. The distribution of protein intensity coefficients of variation are plotted both before (D) and after (E) the scanpy normalization was applied, with distribution of protein CVs plotted across all cells in the entire experiment (left panels) and within each plate batch (right panels).

**
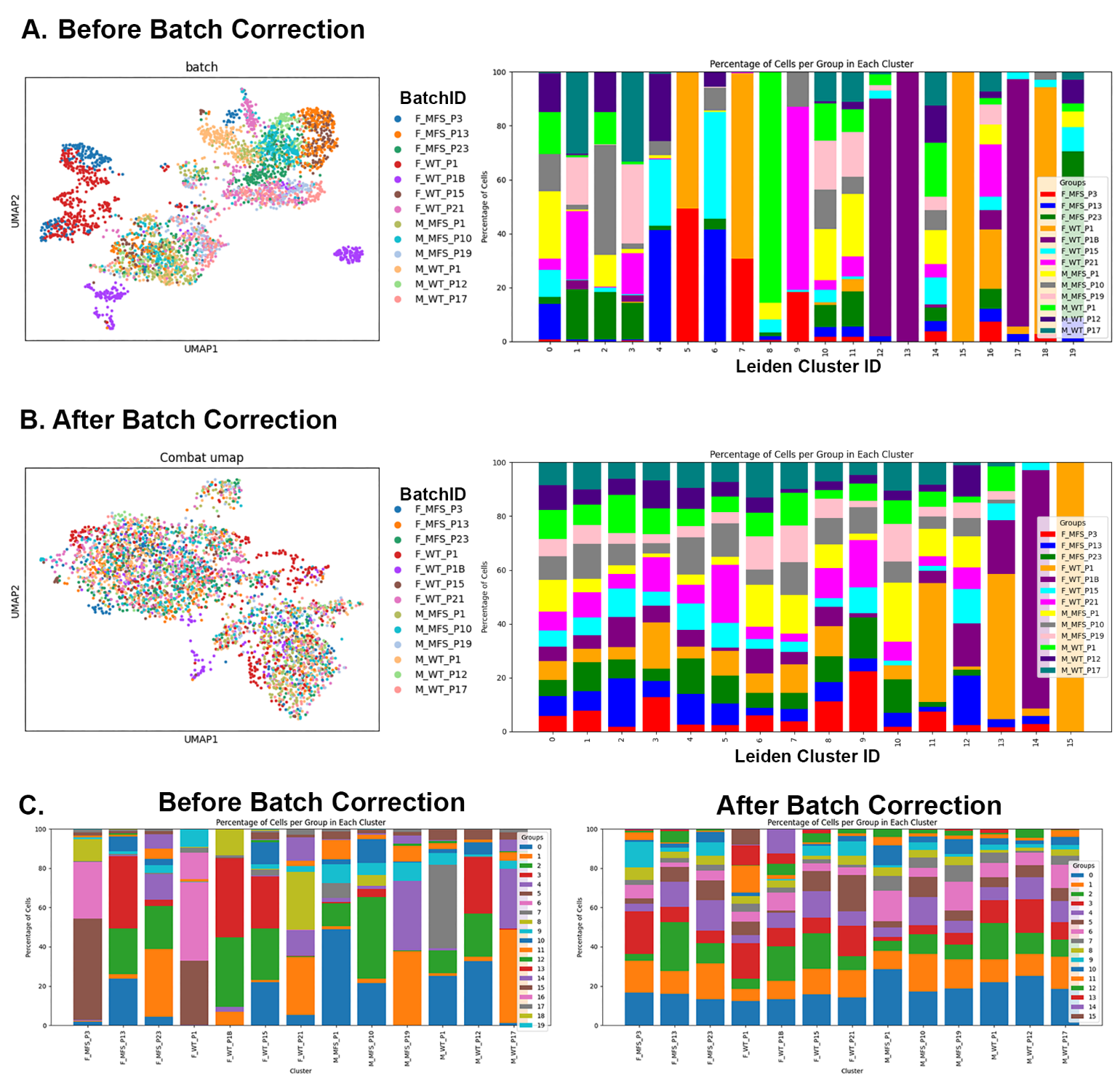
Supplementary Figure 4.** Comparison of cell clustering by plate replicate (e.g., batch) across UMAPs (left panels) and proportion of cells from each replicate assigned to a given leiden cluster (right panels) before (A) and after (B) combat batch correction. A similar comparison of distribution of Leiden clusters, within each batch before and after combat correction, was also performed (C).

**
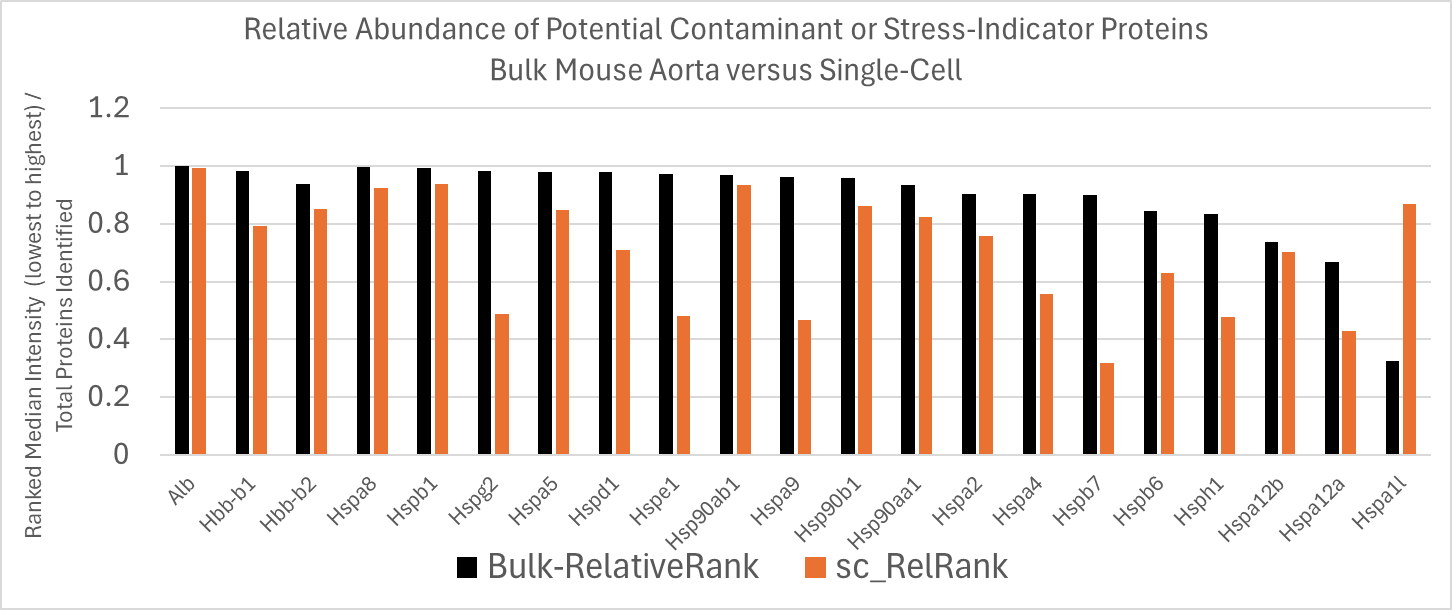

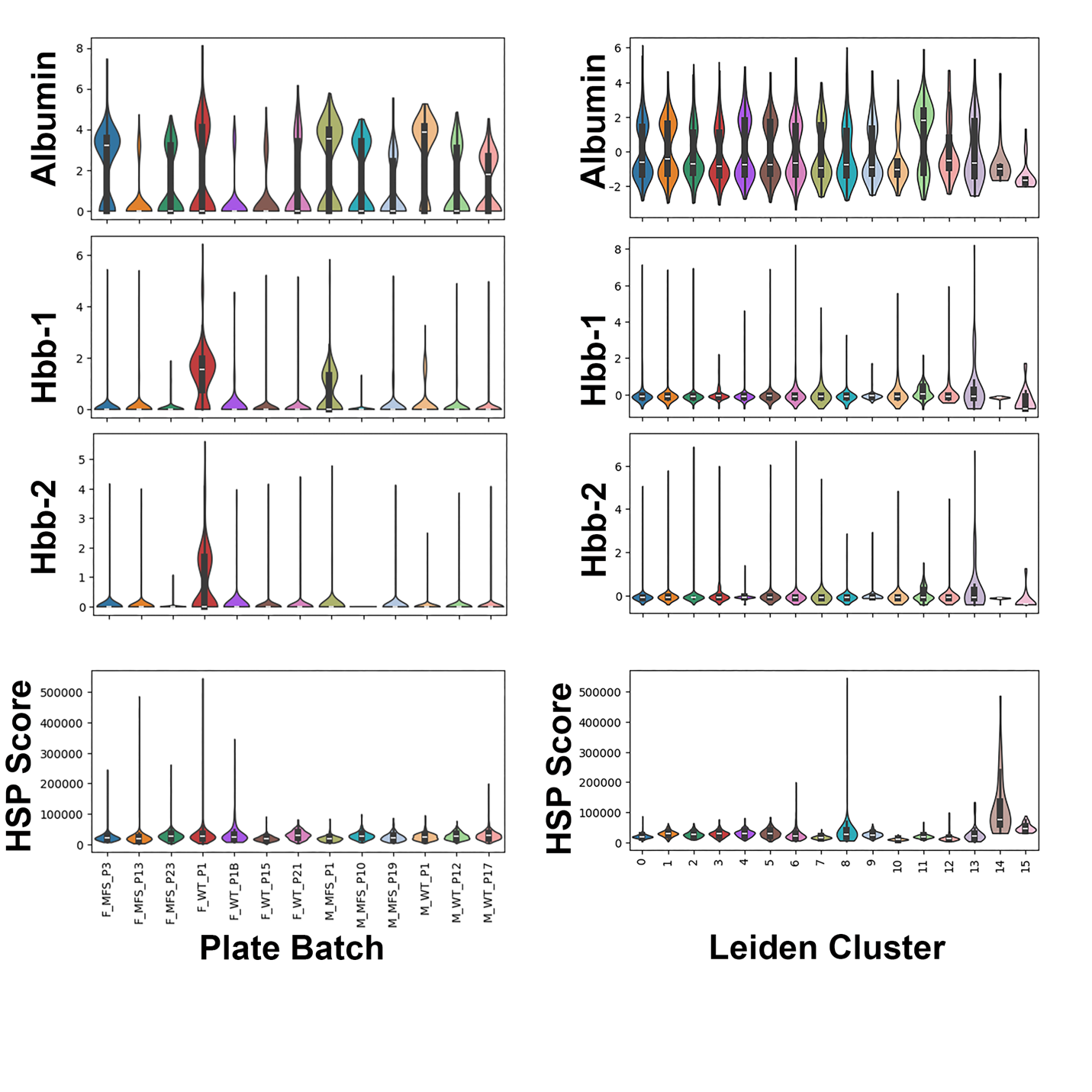
**

**B**

**A**

**Supplementary Figure 5. (A)** The distribution of selected high abundance plasma proteins albumin (Alb), hemoglobin subunits (Hbb-1, Hbb-1) as well as a composite summed heat shock protein score (HSP score) were evaluated across cell isolation plate batches (left panels) and subsequent leiden clusters (right panels). (**B)** The relative ranked abundance of contaminant or possible stress proteins as measured in a dataset of wild-type and MFS mouse bulk aorta proteomes compared to the current single cell dataset.


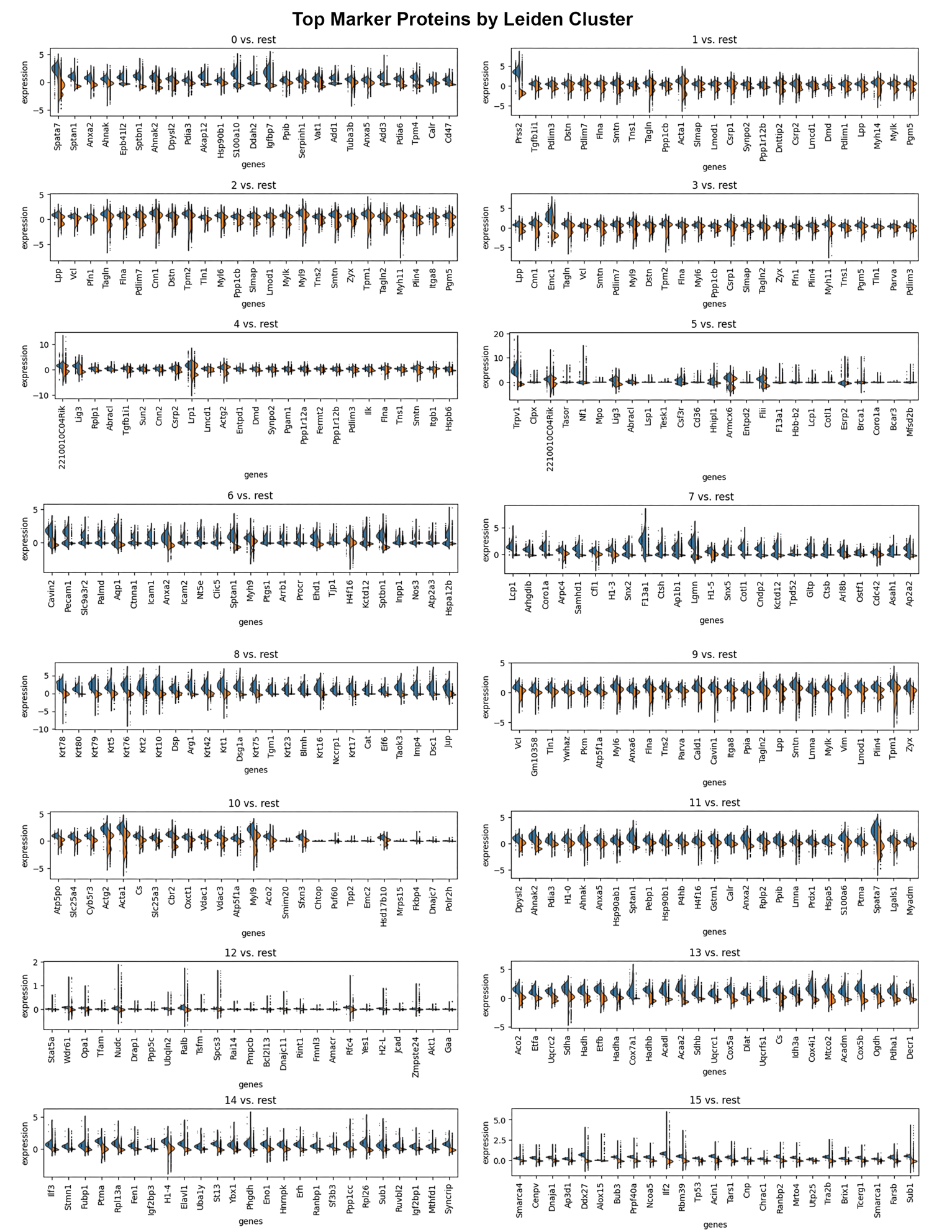
**Supplementary Figure 6.** Comparison violin plots for top markers in each leiden cluster. Blue = cells in cluster, orange = all other cells.

**
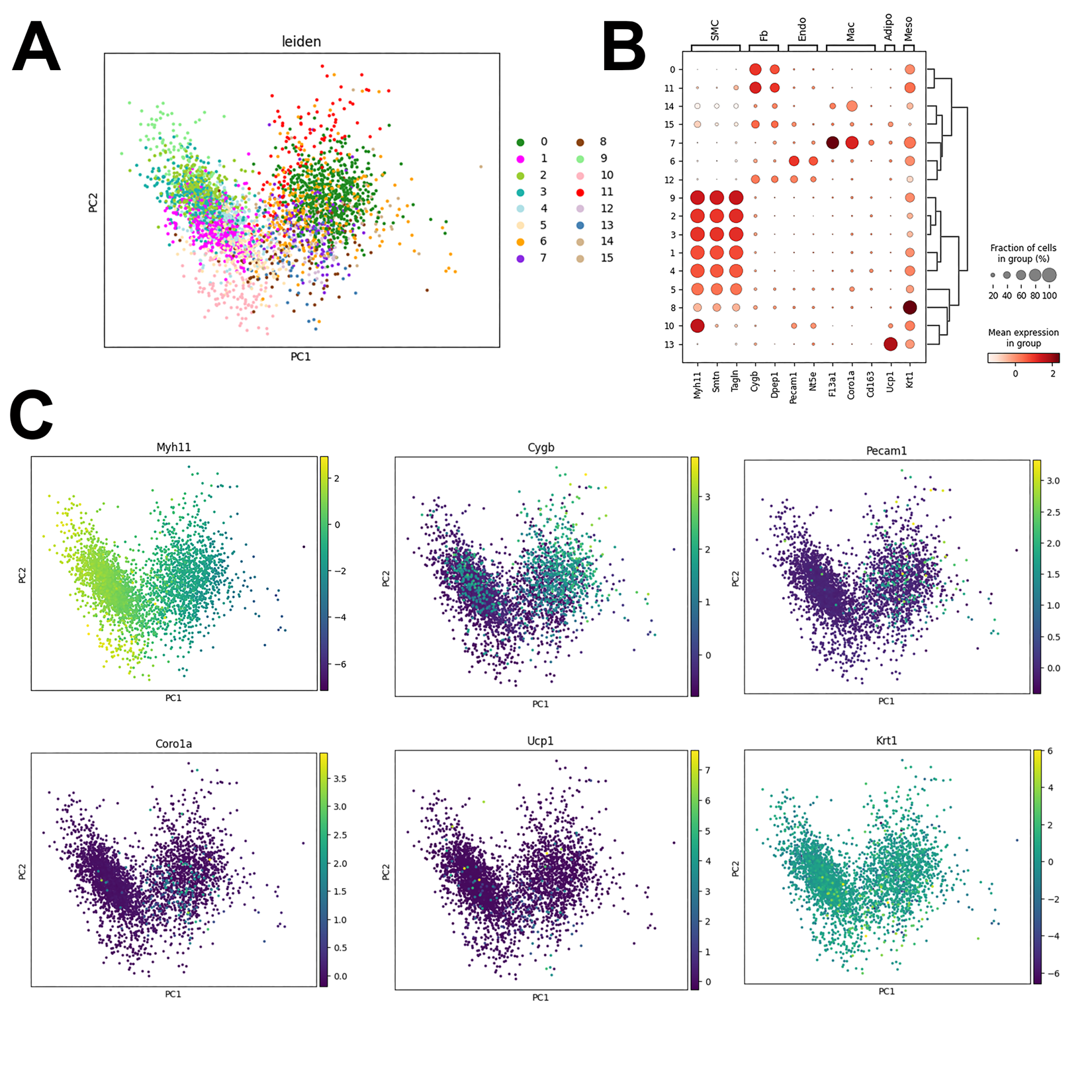
Supplementary Figure 7.** Distrubution of leiden clusters and canonical cell marker proteins across principal components 1 and 2. (A) Leiden cluster assignment distribution across PCs (B) Reciprocal expression profiles of marker proteins and their canonical cell types they represent (C) projected abundance of selected cell type marker proteins by PC1 and PC2.

**Supplementary Figure 8. Distribution of Cell Diameters Across Leiden Designated Proteomic-Cell Clusters.** CellenONE measured cell diameter distributions were plotted according to the leiden-cluster and a priori marker assigned phenotypes. Adipo=Adipocytes; EC= Endothelial Cells; eFC=Endothelial-like Fibroblasts; Fb=Fibroblast; Fb2=Fibroblast type 2; Mac= Macrophage; Meso=Mesothelial; other=unable to assign clear phenotype by canonical markers; SMC1-7=Smooth muscle cell subtypes.

Diameter (µm)


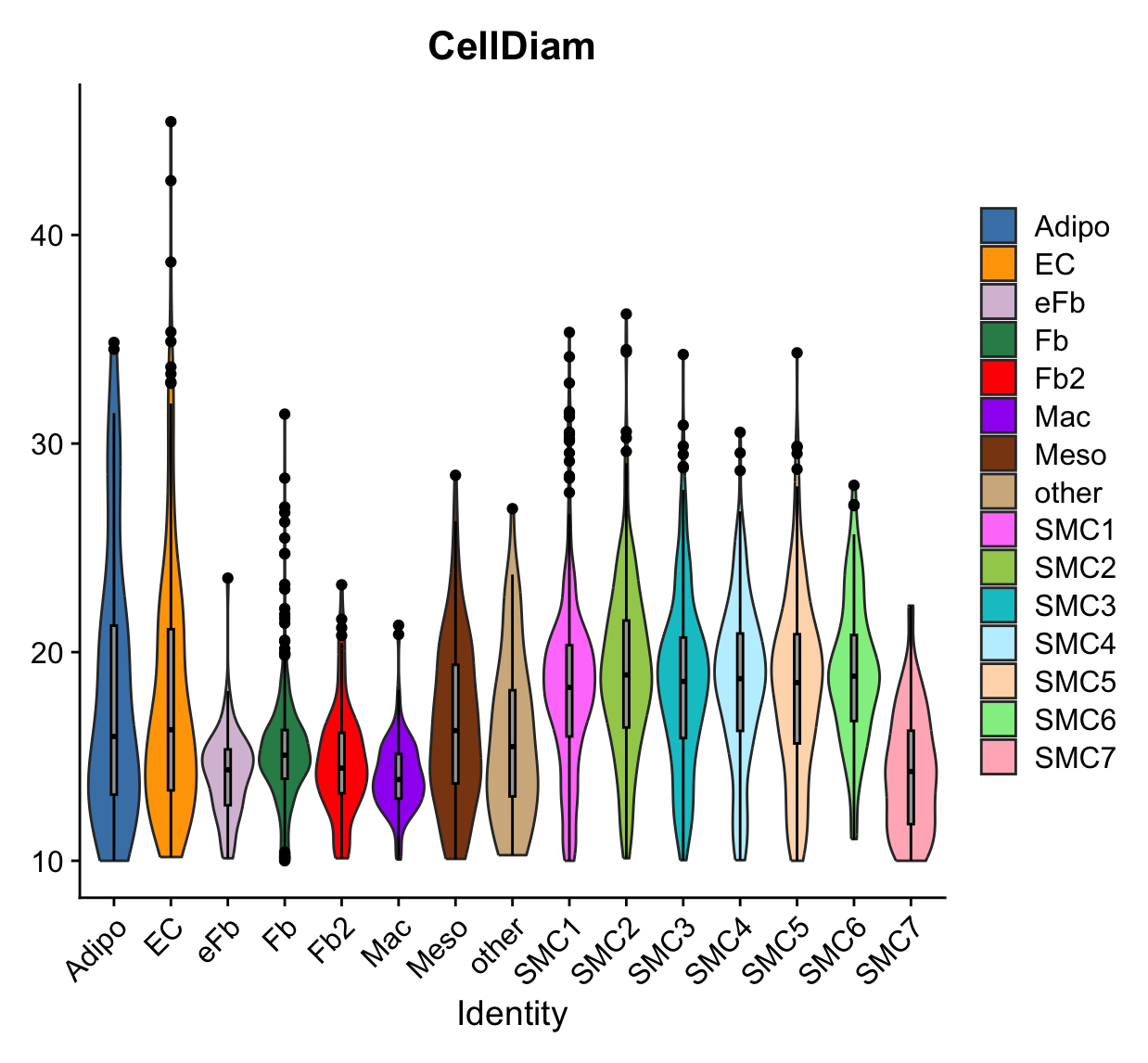


**
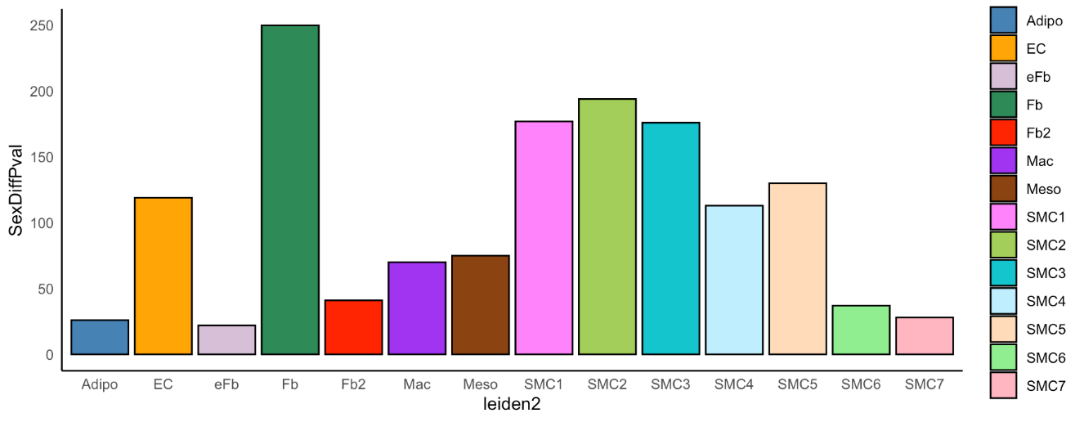

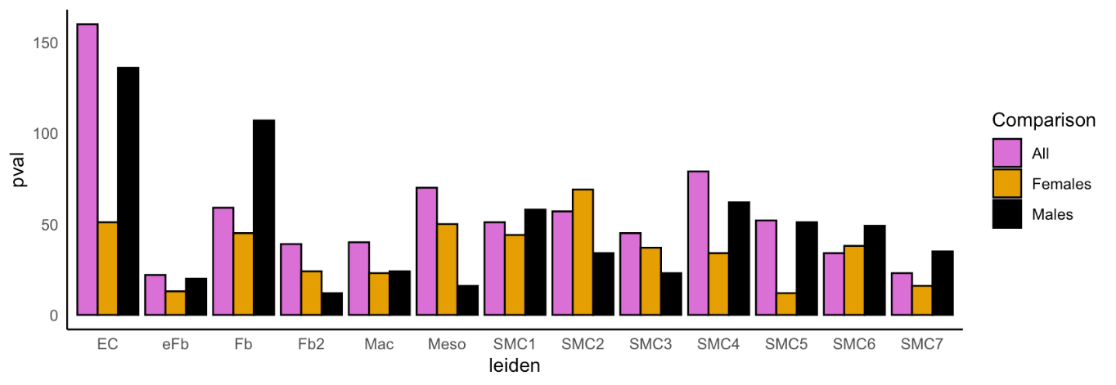
**

**B**

**A**

**Supplementary Figure 9.**  (A) Distribution of proteins with differential expression bewteen Marfan’s syndrome (MFS) and wild-type (WT) across each leiden cluster, according to nominal p value < 0.05 as opposed to the false discovery rate benjamini-hochberg adjusted values. (B) Distribution of proteins with differential expression bewteen Male and Female mice across each leiden cluster, according to nominal p value < 0.05 as opposed to the false discovery rate benjamini-hochberg adjusted values.

**Supplementary Figure 10.
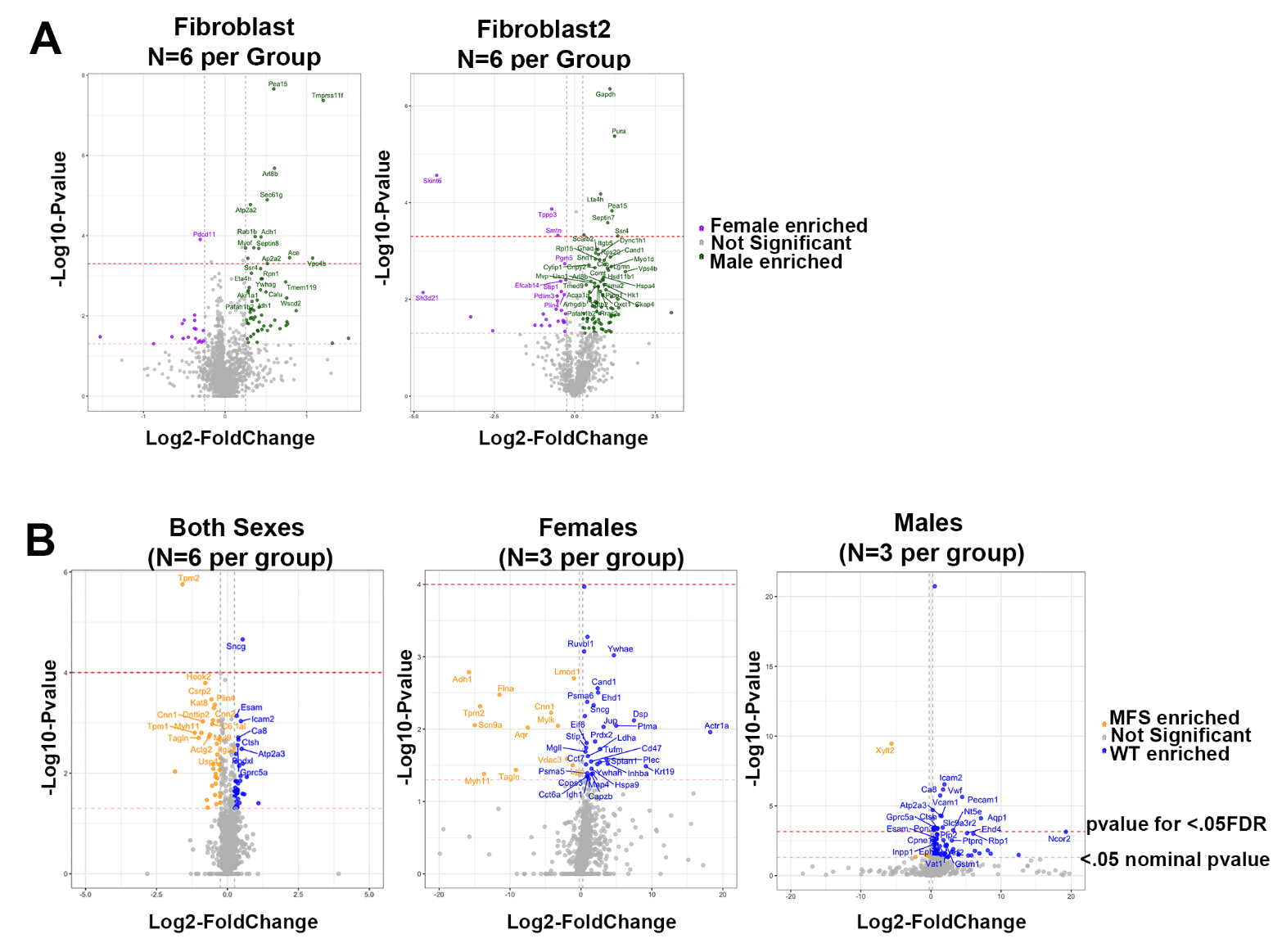
Selected Volcano Plots to Visualize Patterns of DEP within Cell Clusters.** (A) Volcano plots were generated for the two Fibroblast cell clusters, visualizing the Log2-Fold Change relative to negative-log (base 10) p-value for proteins as compared between male- and female-derived cells. N=6 per group (ignoring cell genotype), statistics calculated using linear mixed effects modeling. (B) Volcano plots were generated for the two Fibroblast cell clusters, visualizing the Log2-Fold Change relative to negative-log (base 10) p-value for proteins as compared between MFS and WT derived endothelial cells, first collapsing both sexes into a single group (left-most panel), and then separating the comparison within females (middle panel) and males (right panel). In both sets of volcano plots (A & B), a dark pink dashed line along intercepts the y-axis at the point corresponding to the FDR < 0.05, whereas a lighter pink line intercepts the y-axis a p<0.05. Grey dotted lines intercept the x-axis at an absolute Log2 fold change of 0.3.


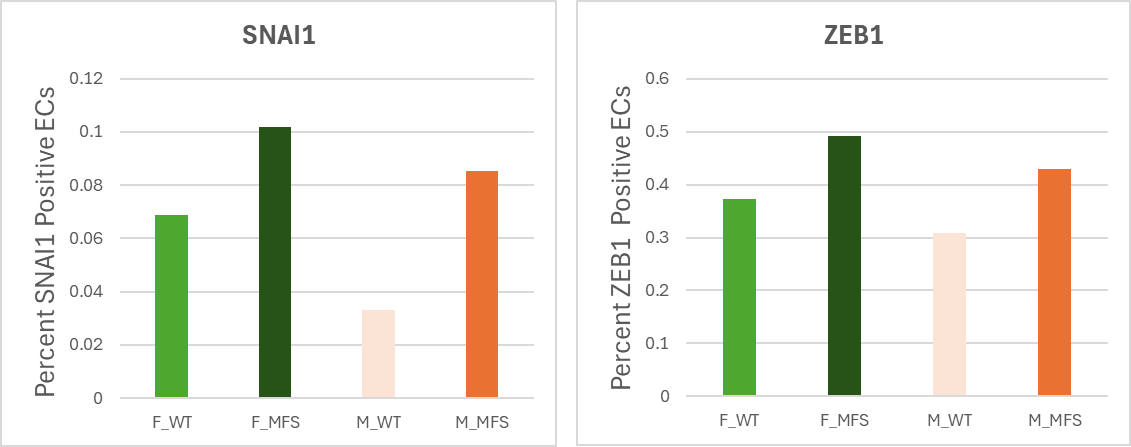


**Supplementary Figure 11.** Percentage of *Snai1* and *Zeb1* positive endothelial cells quantified from a separate, single-cell RNA sequencing dataset produced from the aortic root of the Marfan *Fbn1^C1041G/+^* mouse. Specifically, the number of *Zeb1* and *Snai1* positive cells within the two endothelial cell (EC) clusters (Leiden 9 and 11) were counted and analyzed relative to the total number of ECs per genotype from our reanalysis of the Pedroza et al Marfan mouse single-cell transcriptomics study.

**
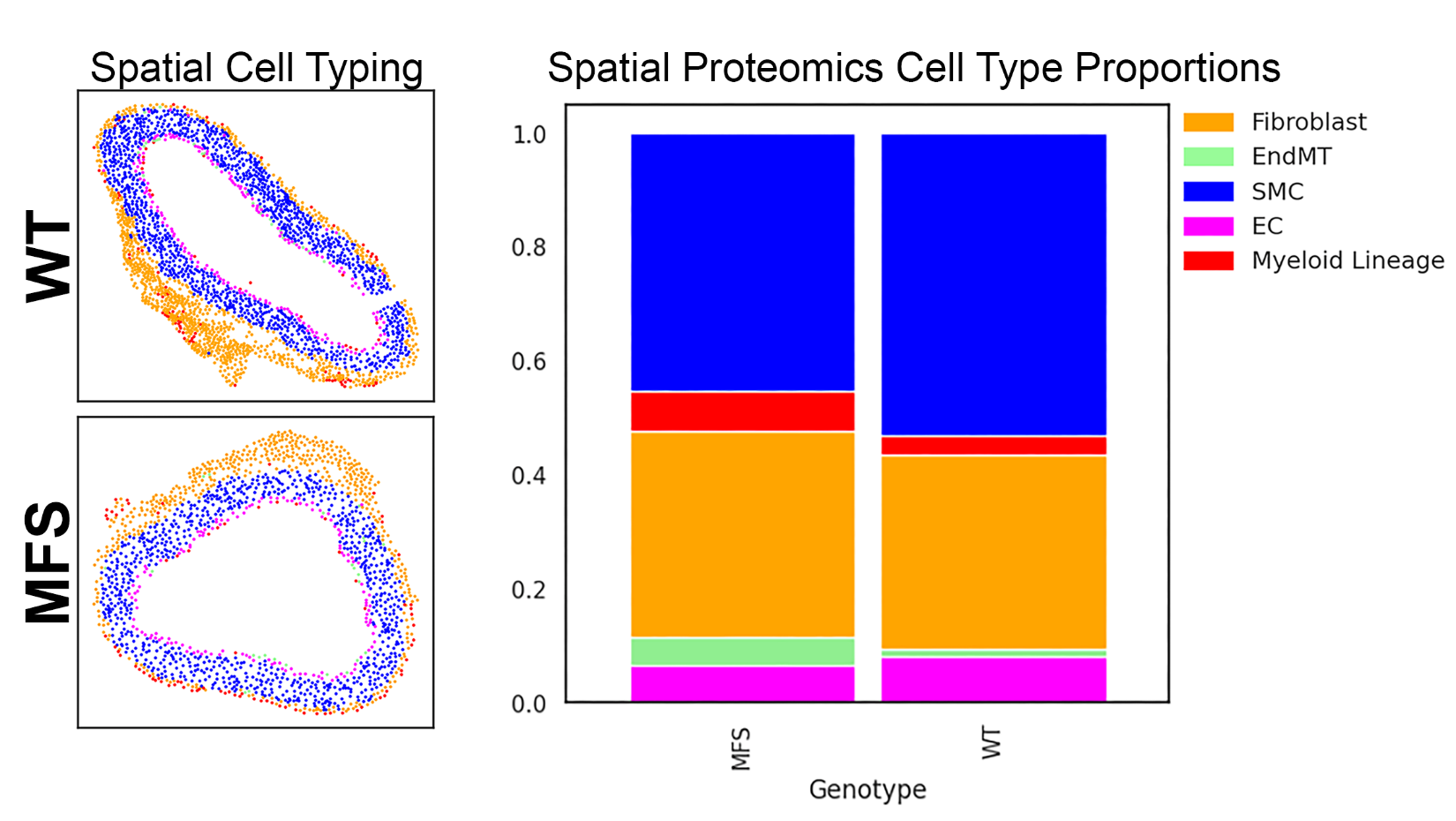
**

**Supplementary Figure 12. Overall Results from the Lunaphore Comet Spatial Proteomis Analysis.** Left Panel: Representative images showing projections of the centroided x and y coordinates of StarDist segmentation estimated cells, with colors overlaid based on the canonical-marker based phenotyping. Right Panel: Relative proportions of each general cell type annotated between MFS (N=2) and WT (N=2) mice.


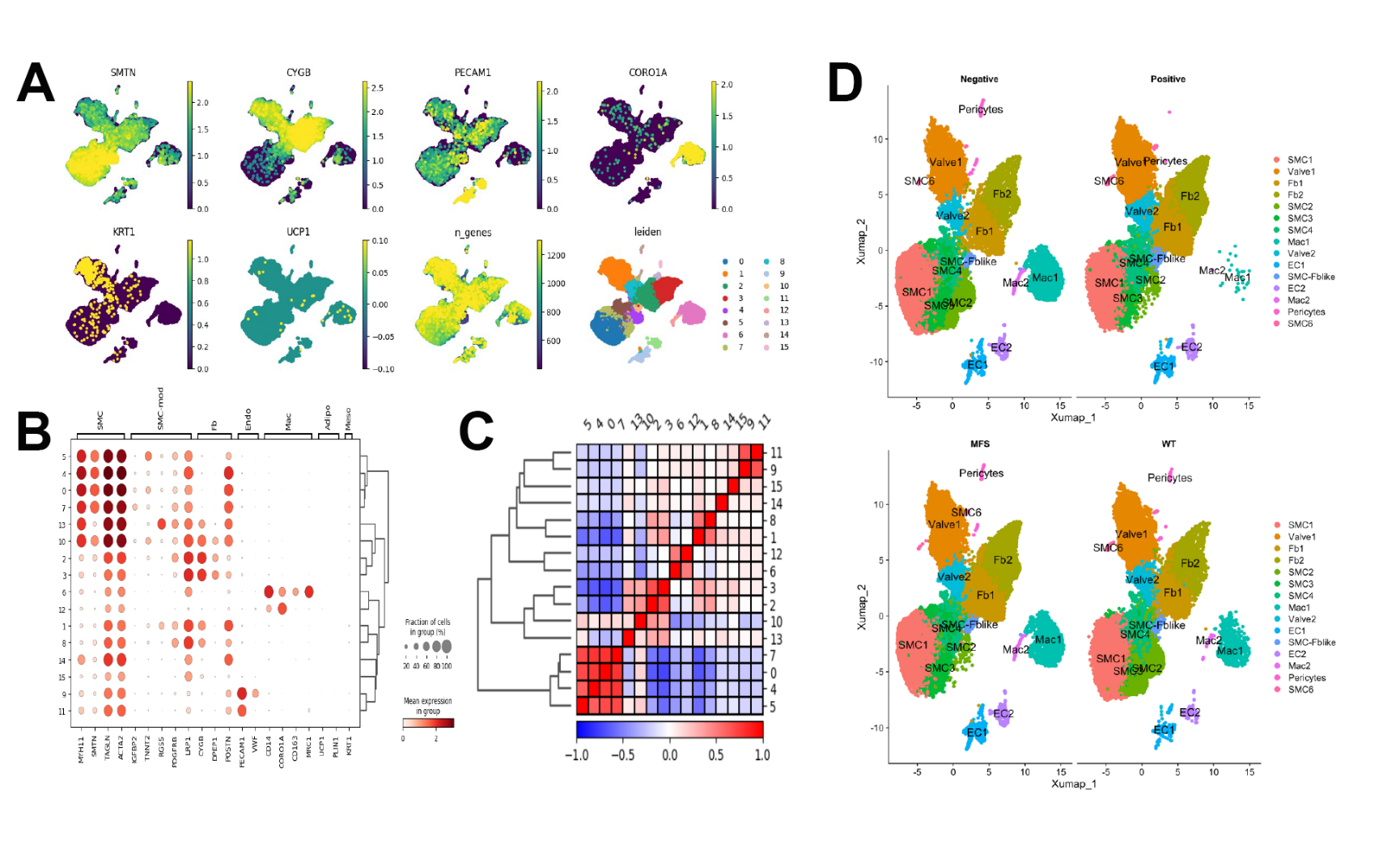
**Supplementary Figure 13.** Results of the Scanpy reanalysis of Pedroza et al. single cell transcriptomic data performed on Marfan’s Syndrome (MFS) and wild-type (WT) aorta with and without second heart field (SHF)-embryonic lineage tracing. (A) Cell type marker expression pattern across Scanpy-identified leiden clusters. (B) Marker dot plots by cell type. (C) Cell type correlelogram. (D) Comparison density plots of cells by leiden cluster divided according to lineage tracing positive / negative (upper panel) or genotype (lower panel).


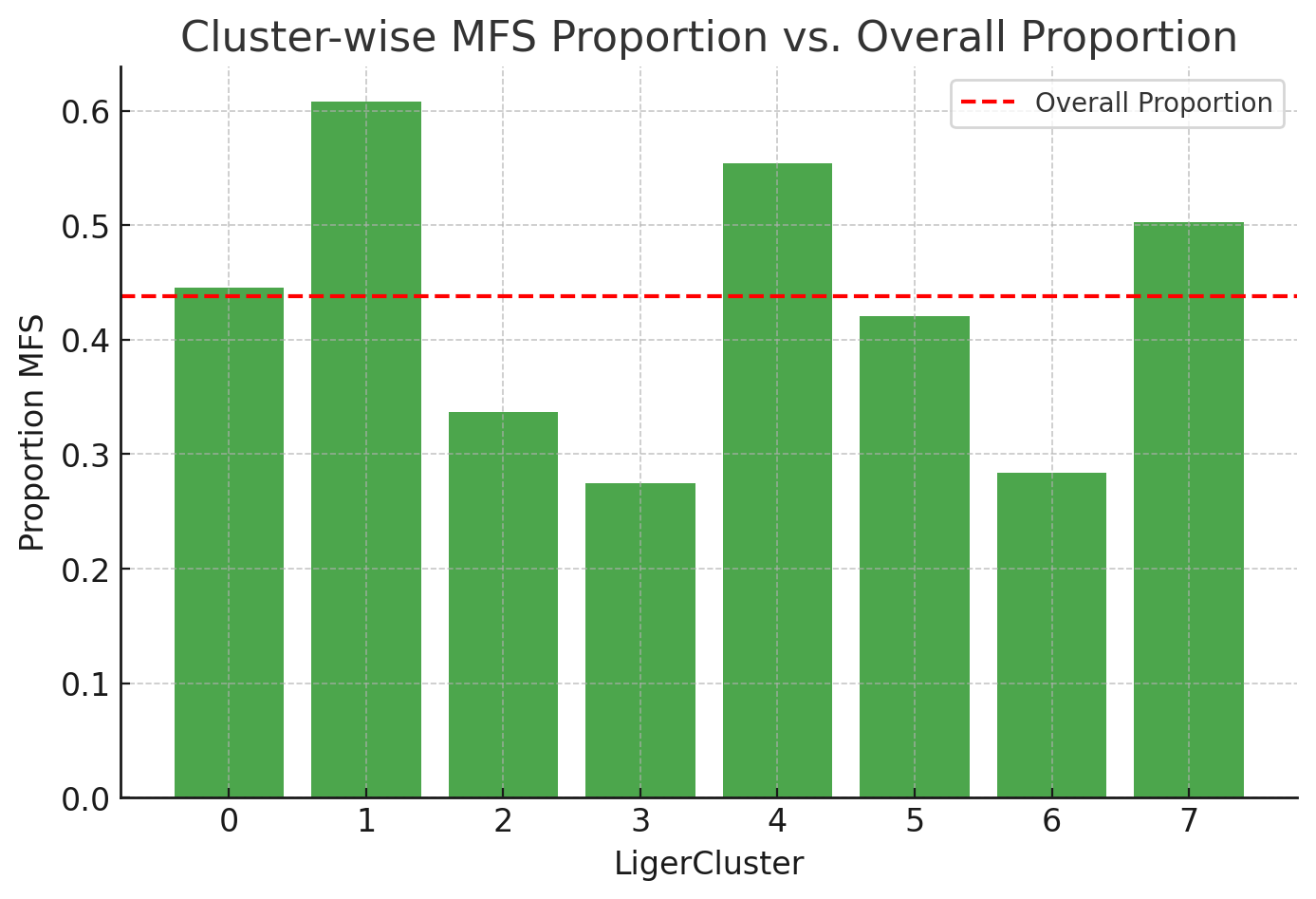

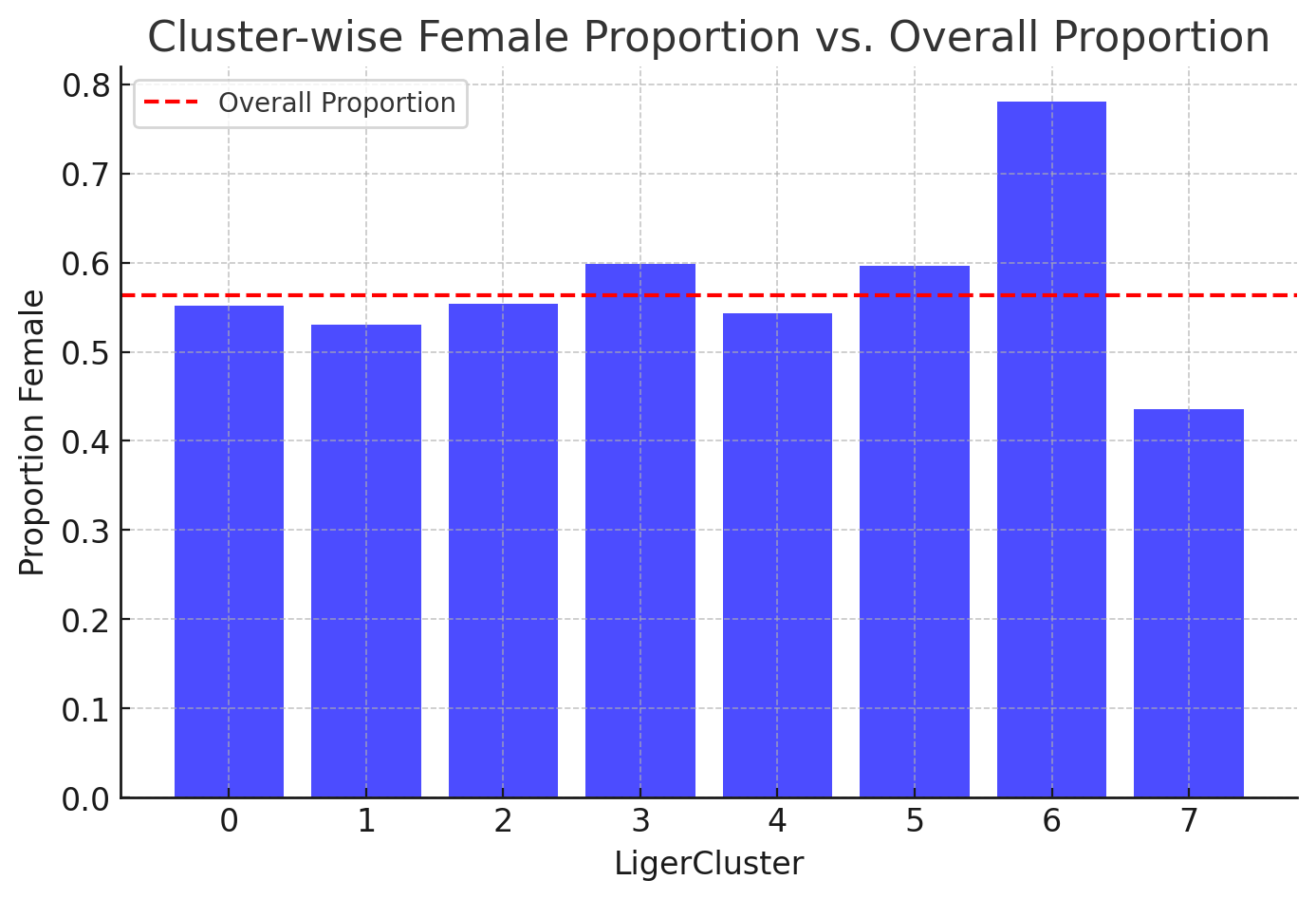


*

*

*

*

*

*

*

**Supplementary Figure 14.** Statistical analysis comparing proportion of Female (Top Panel) and Marfan’s Syndrome (MFS) (Bottom Panel) cells within each cluster, relative to the global proportion of cells from these two categories. Asterisks indicate clusters with statistically significant deviation of observed proportion of the focus cell type (p < 0.05).


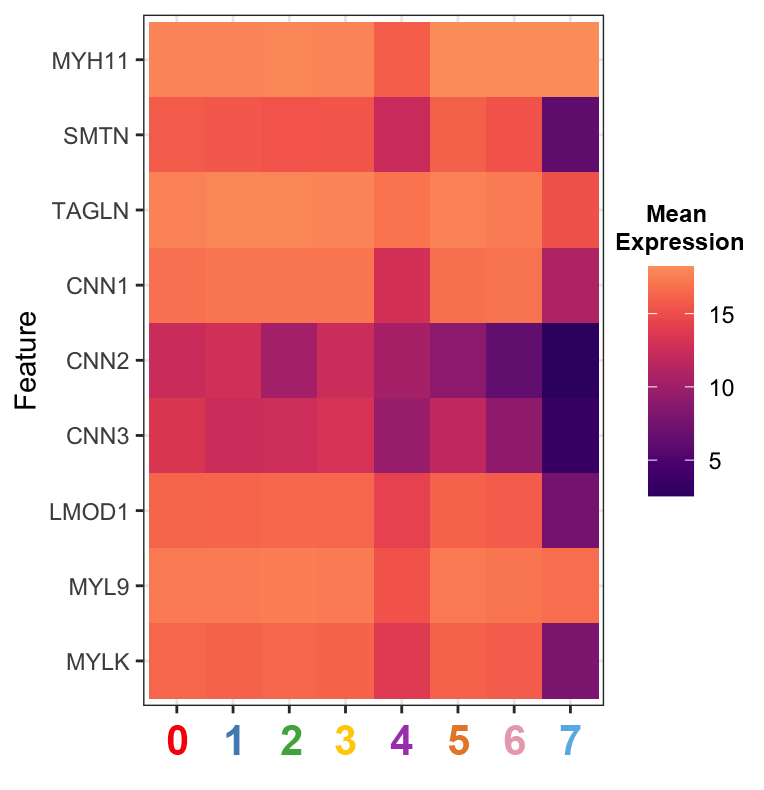


**Supplementary Figure 15.** Mean Multiomic Expression level of canonical smooth muscle cell (SMC) contractile markers across leiden cell clusters. Mean expression is calculated from normalized, scaled integration by the LIGER analytical tool. MYH11 indicates Myosin Heavy Chain 11; SMTN, Smoothelin; TAGLN, Transgelin; CNN1/2/3, Calponin 1/2/3; LMOD1, Leiomodulin 1; MYL9, Myosin light chain 9; and MYLK, Myosin Light Chain Kinase.


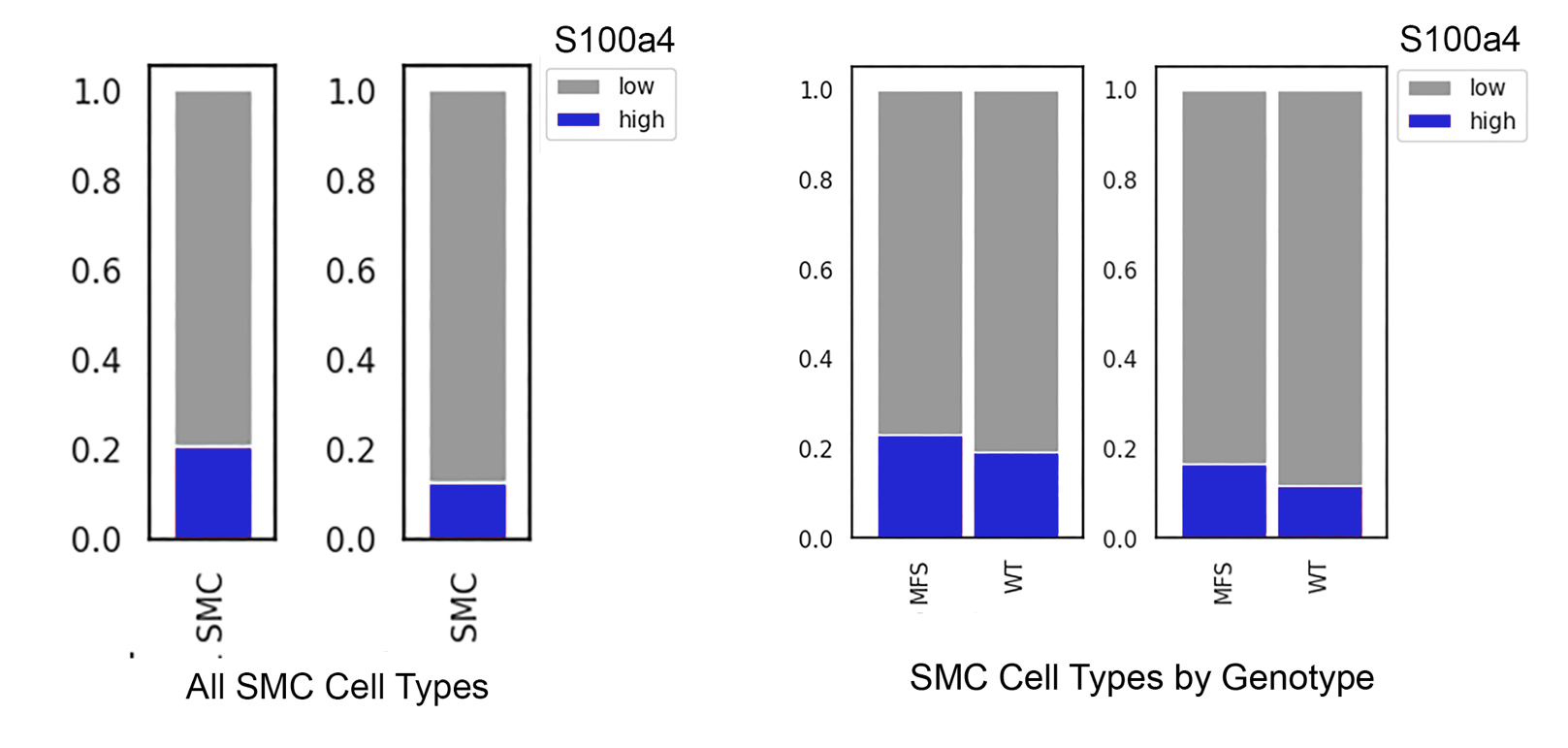


Supplementary Figure 19. Total proportion of S100a4 positive cells compared between SCPMS and Spatial Proteomic datasets. The total count of S100a4-positive SMCs was quantified between SCPMS and lunaphore datasets, demonstrating comparable levels of ~20% of all SMCs with high S100a4 (left panel) and only slightly elevated S100a4 levels in Marfan (MFS) relative to wild-type (WT) mice in both datasets (right panel).

**Supplementary References**

1. A. T. Kong, F. V. Leprevost, D. M. Avtonomov, D. Mellacheruvu and A. I. Nesvizhskii, Nat Methods **14** (5), 513-520 (2017).

2. V. Demichev, C. B. Messner, S. I. Vernardis, K. S. Lilley and M. Ralser, Nat Methods **17** (1), 41-44 (2020).

3. F. A. Wolf, P. Angerer and F. J. Theis, Genome Biology **19** (1), 15 (2018).
